# Overexpression of TMEM79 combined with SMG5 is related to prognosis, tumor immune infiltration and drug sensitivity in hepatocellular carcinoma

**DOI:** 10.1186/s40001-023-01388-w

**Published:** 2023-11-07

**Authors:** Yu Wang, Qin Jin, Shu Zhang, Yan Wang

**Affiliations:** 1 Department of Pathology, Affiliated Hospital of Nantong University, Nantong, China; 2 Medical School of Nantong University, Nantong, China

**Keywords:** TMEM79, SMG5, HCC, Prognosis, Immune infiltration

## Abstract

**Background:**

Hepatocellular carcinoma (HCC) is a primary liver malignancy that is now relatively common worldwide. TMEM79 has been reported to play diagnostic and prognostic markers in a variety of cancers and was found to be closely associated with immune infiltration. SMG5 is associated with immune cell infiltration in HCC. Multiple nonsense-mediated mRNA processes require the involvement of SMG5. TMEM79 and SMG5 complexes may be prognostic markers for prostate cancer. However, the relationship between TMEM79 expression in HCC and prognosis, its role and mechanism of action, and its relationship with SMG5 have not been studied. This article focuses on not only the prognostic role of TMEM79 and its biological significance, including immuno-infiltration, tumor mutations and drug sensitivity, but also the interaction with SMG5 in HCC.

**Methods:**

Differential expression analysis and the multiCox proportional hazards regression analyses of TMEM79 and SMG5 were performed by multiple databases. Then, use IHC to verify our results. Subsequently, we used R software to analyze the clinical phenotype of both: analysis of clinicopathological features, enrichment analysis, analysis of immune infiltration, analysis of immune checkpoints, analysis of drug sensitivity, and immunotherapy.

**Results:**

Both the database studies and the results of our research group showed that TMEM79 and SMG5 were differentially expressed in HCC and normal tissues. Validation of immunohistochemistry showed that differential expression of TMEM79 and SMG5, which influenced the prognosis of patients with HCC, could be an independent prognostic factor. Results of the TCGA database study showed that TMEM79 and SMG5 were correlated with immune infiltration, immune checkpoints, drug sensitivity, and immunotherapy. We typed TMEM79-related molecules in HCC according to R software. Two types of TMEM79 correlated with clinical features, survival of patients with HCC, and immune infiltration.

**Conclusion:**

TMEM79 are highly expressed in HCC and play an important role in the prognosis of patients with HCC. TMEM79 and SMG5 are positively correlated and may both associated with immune infiltration, and closely linked to immune checkpoints, drug sensitivity, and immunotherapy in HCC.

## Background

Environment surrounding the tumor is comprised of a diverse population of immune cells, endothelial cells, and fibroblasts. The structure of the TME (The tumor microenvironment) has been demonstrated to impact the efficacy of immune-checkpoint blockade (ICB), which employs the infiltration of immune cells within tumors to revitalize a potent anti-cancer immune reaction [1]. Today, scientists can design therapeutic agents at the cellular molecular level to target well-defined oncogenic sites and cause tumor cell-specific death. Therefore, we need to discover more immune checkpoints to reactivate the immune activation and prevent tumor progression and metastasis.

The incidence of HCC is increasing year by year, making it the sixth most prevalent form of cancer across the globe [2]. Hepatocellular carcinoma (HCC) is the most common primary cancer of the liver and accounts for 90% of hepatic cancers [3]. Despite advances in medical, locoregional and surgical therapies, HCC remains one of the most common causes of cancer-related death globally [4]. The use of immune checkpoint inhibitors has been shown to produce meaningful improvements in survival time in patients with HCC [5].

TMEM79 is a protein-encoded gene that is expressed in membranes in squamous epithelial and prostate gland cells and contributes to epidermal integrity and skin barrier function. TMEM79 has been linked to both dermatitis and atopic dermatitis as potential health concerns. High expression of TMEM79 plays a role in promoting many cancer types, and it has been studied that TMEM79 is a diagnostic marker for prostate cancer [6]. TMEM79 has been reported to be associated with immune cell infiltration in prostate cancer [7]. TMEM79 has been found to be a possible diagnostic marker for prostate cancer. It has been found to distinguish between benign and malignant prostate tissue. TMEM79 may be associated with the clinical progression stage of colorectal cancer. It may be a diagnostic marker for metastatic malignant melanoma. It is involved in the immune response during the metastasis process of malignant melanoma and is related to immune cells. However, the underlying mechanisms of HCC remain largely unknown despite extensive research into its pathology. We found in our study of the reciprocal protein of TMEM79 that it interacts with SMG5, and SMG5 was closely related to TMEM79 by cBioportal (cBioPortal for Cancer Genomics).

SMG5 is a kind of RNA-binding proteins (RBPs). In addition, they have been shown to be key regulators of oncogenesis and tumor progression [8]. SMG5 plays a role in the degradation of mRNA through the process of nonsense-mediated decay and encodes a protein. SMG5 has been linked to the development of pancreatic cancer and epiphyseal chondrodysplasia among various illnesses [9]. Which is associated with prognostic effects through mutational actions in HCC [10]. SMG5 may be associated with a poorer prognosis in gastric cancer [11]. According to reports, SMG5 may be a high-risk factor for HCC prognosis [12]. SMG5 has extensive effects on the proliferation, survival, and tumor growth of HCC cells. SMG5 promotes HCC cell proliferation and tumor growth. According to the study, SMG5 is associated with the infiltration of immune cells, such as macrophages, B cells, and T cells in HCC [10].

In this paper, we focus on expressions of TMEM79 and SMG5 in HCC and how they affect the prognostic role in HCC. Next, we investigated the interaction between TMEM79 and SMG5, as well as their expression in HCC patients, their prognostic impact on HCC patients, and functional phenotype analysis.

## Results

### The expression of TMEM79 in HCC in the TCGA database

**Fig. 1:**
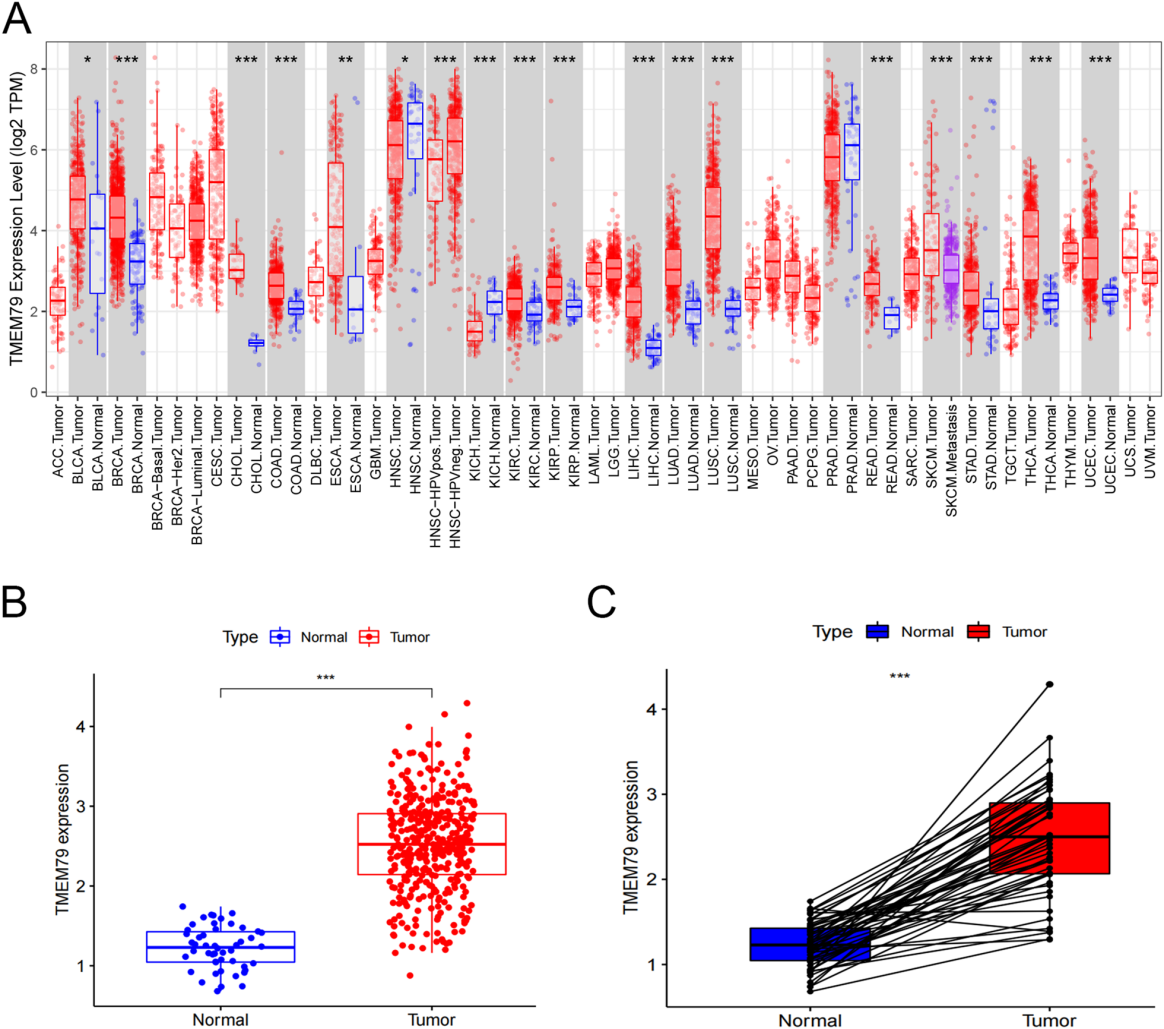
Expression of TMEM79 in HCC from TCGA database. **A** Expression levels of TMEM79 in various cancers by TCGA database. **B**, **C** Expression of TMEM79 in 374 HCC tissues and 50 adjacent tissues. *P < 0.05, **P < 0.01 and ***P < 0.001

To analyze the expression levels of TMEM79 in pan-cancer and determine its mRNA expression profile, TIMER2.0 (https://link.zhihu.com/?target=http%3A//timer.comp-genomics.org/) database was employed. Expression of TMEM79 was found to be increased in 15 types of cancer in pan-cancer tissues, comprising uroepithelial carcinoma of the bladder, breast cancer, bile duct cancer, colon cancer, esophageal cancer, renal clear cell carcinoma, renal papillary cell carcinoma, hepatocellular cancer, lung adenocarcinoma, lung squamous carcinoma, rectal adenocarcinoma, melanoma, gastric cancer, thyroid cancer, and endometrial cancer. On the other hand, the levels of TMEM79 were reduced in three types of cancer, consisting of head and neck squamous cell carcinoma, renal suspicious cell carcinoma, and prostate cancer (Fig. 1A). To investigate the mRNA expression level of TMEM79 in HCC. TCGA database (https://portal.gdc.cancer.gov/repository) was used significantly elevated levels of TMEM79 mRNA expression were observed in HCC compared to normal tissues. (Fig. 1B, C).

### Impact of TMEM79 on survival and prognosis in HCC in databases

**Fig. 2:**
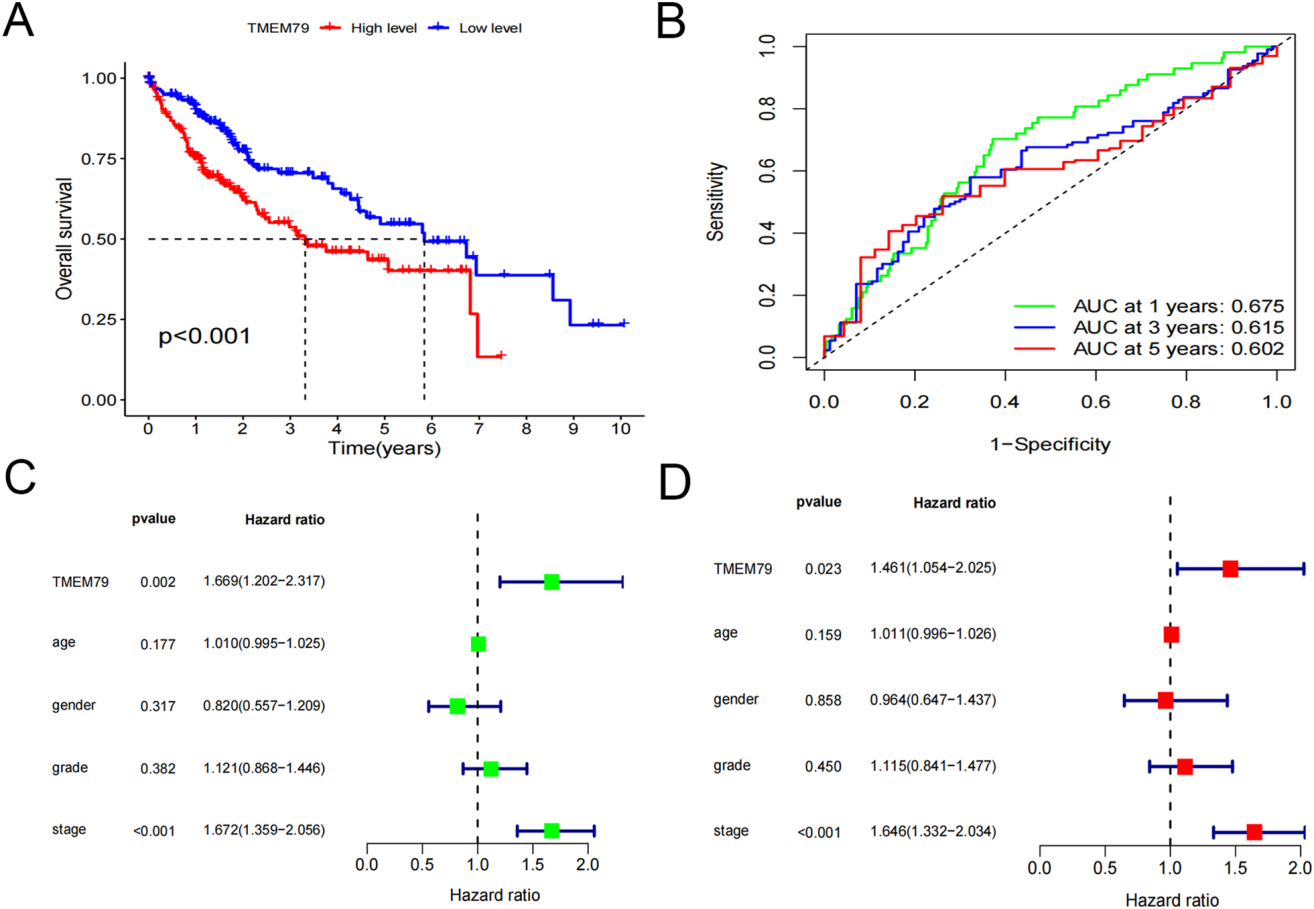
Prognostic significance of TMEM79 in HCC in the TCGA database. **A** Kaplan–Meier overall survival curve for patients with HCC based on tumor expression of TMEM79. **B** 1-Year, 3-year, and 5-year time-dependent subject workup characteristics curves for predicted values of TMEM79 on overall survival. **C**, **D** MultiCox proportional hazards regression analyses of TMEM79

Four hundred and twenty-four patients with clinical data were included in the survival analysis and the patients with case HCC were divided into a high TMEM79 expression group and a low TMEM79 expression group according to the median critical value. The survival analysis results indicated that patients in the low expression group had a significantly longer overall survival (OS) compared to those in the high expression group (Fig. 2A). The area under the curve (AUCs) for the TMEM79-expressing group of OS were 0.675, 0.615, and 0.602 at 1 year, 3 years, and 5 years, respectively (Fig. 2B). Univariate and multivariate analyses of TMEM79 showed that TMEM79 was an independent prognostic factor (Fig. 2C, D).

### Analysis of TMEM79-related derivatives in the TCGA database

**Fig. 3:**
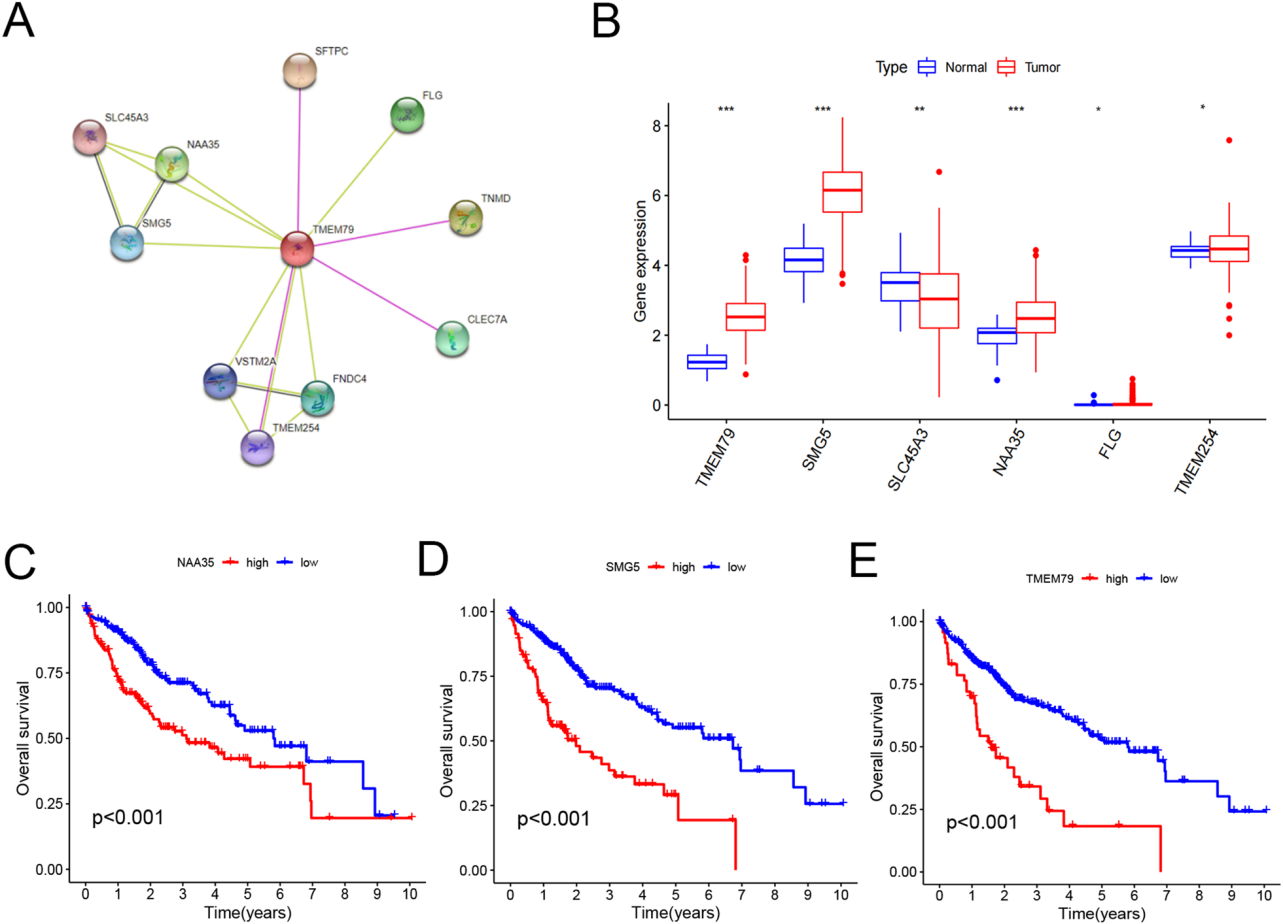
Analysis of correlation molecules of TMEM79. **A** Analysis of TMEM79-interacting proteins and derived factors. **B** Differential expression of TMEM79-related factors. **C**, **D**, **E** Prognostic survival analysis of NAA35, SMG5 and TMEM79. (*P < 0.05, ***P < 0.005)

TMEM79-related interacting proteins were analyzed using the string database, followed by further analysis of 10 genes (Fig. 3A). The expression levels ofTMEM79-related-derived factors in 374 liver cancer patients and 50 normal human liver tissues were examined on the TCGA database (Fig. 3B). The molecules that were differentially expressed in tumor and normal tissues by R software analysis were TMEM79, SMG5, NAA35, SLC45A3, FLG, TMEM254. The findings indicated that in tumor tissue when compared to normal tissue, six factors, among them TMEM79, were expressed differently. Prognostic analysis of these six genes showed that NAA35, SMG5, and TMEM79 have a prognostic role in HCC, and the higher the expressions, the worse the prognosis (Fig. 3C–E). Then, FLG, SLC45A3and TMEM254 have no significant impact on the prognosis and survival time of HCC patients. One study found that SMG5 was reported to have a prognostic role in HCC [12].Therefore, we next analyzed the expression and prognostic value of SMG5 in HCC.

### Expression and prognostic value of SMG5 in HCC in TCGA database

**Fig. 4:**
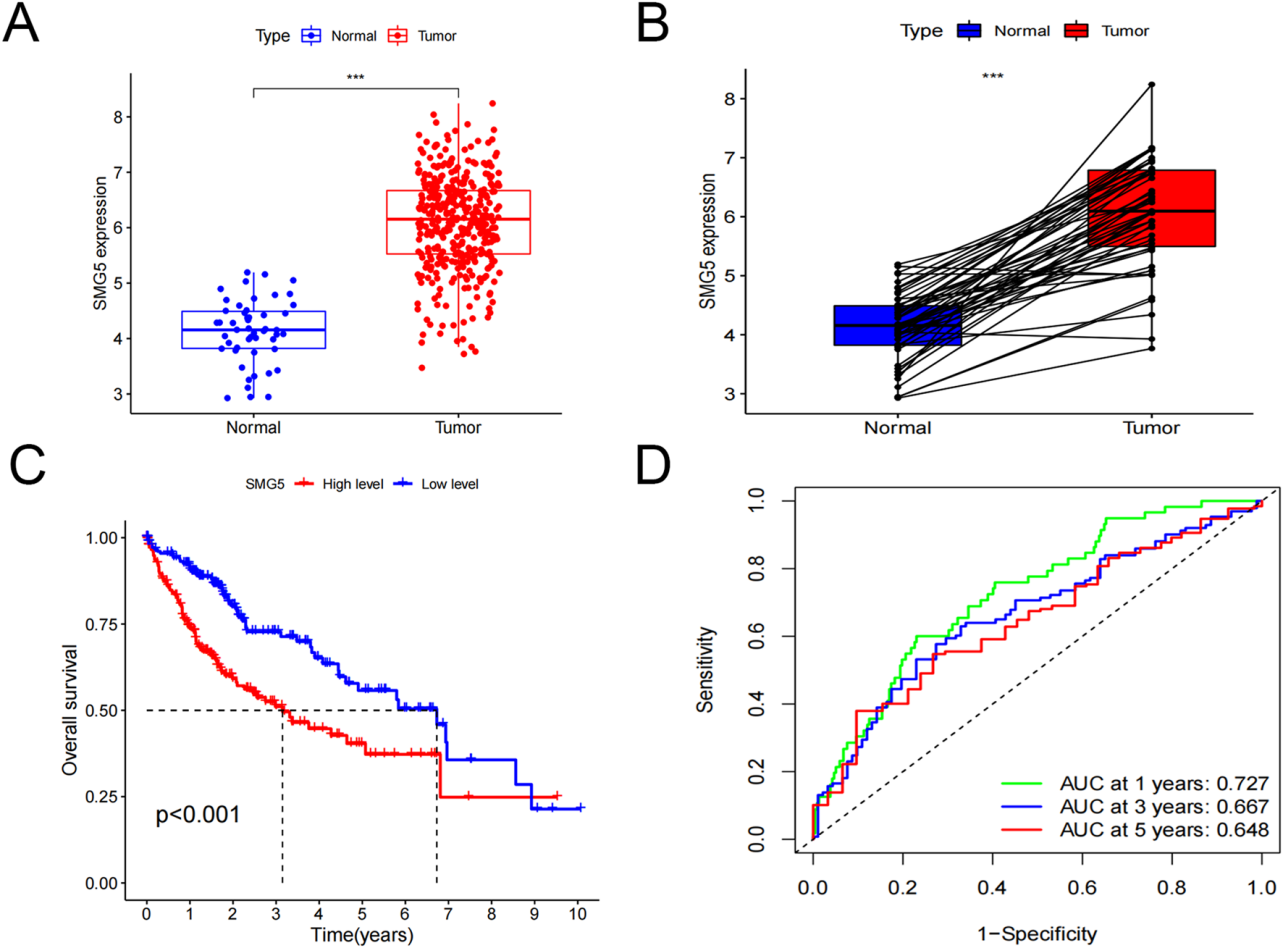
Expression and prognostic value of SMG5 in HCC. **A**, **B** Analysis of the difference between SMG5 in HCC and normal tissue. **C** Relationship between overall survival of SMG5 in HCC. **D** Prognostic ROC curve of SMG5 in HCC. *P < 0.05, ***P < 0.005

mRNA expression of SMG5 in HCC was investigated using the TCGA database. A significantly higher expression level of SMG5 mRNA was observed in LIHC compared to normal tissues (Fig. 4A, B). Survival analysis showed that the OS was significantly longer in the low expression group than that in the high expression group (Fig. 4C). The area under the curve (AUCs) of the SMG5 expression group of OS at 1, 3, and 5 years were 0.727, 0.667, and 0.648 (Fig. 4D).

### Expressions of TMEM79 and SMG5 and their correlation in HCC in our research

**Fig. 5:**
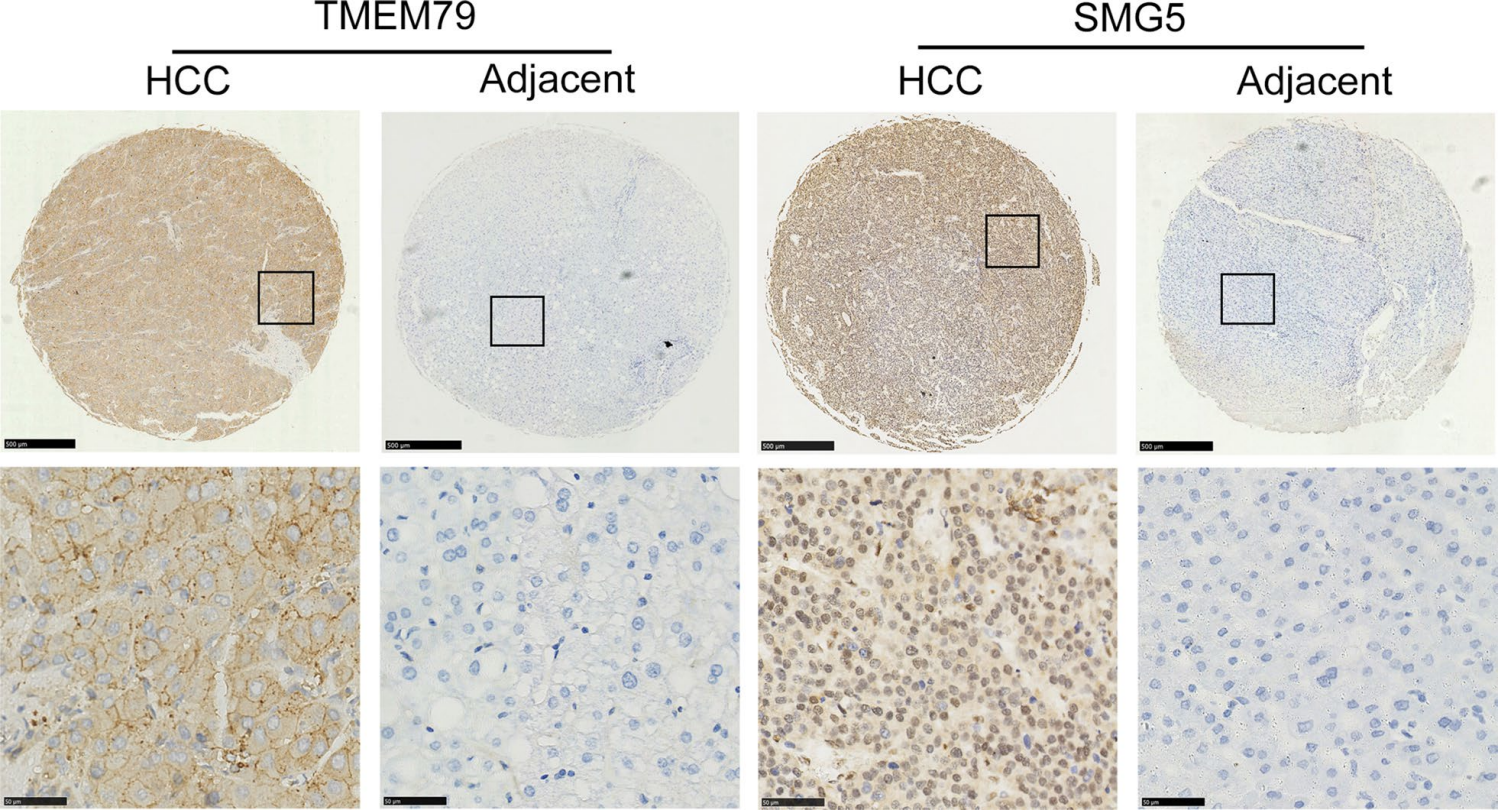
Expressions of TMEM79 and SMG5 proteins in HCC and adjacent normal tissues by IHC (magnification 4 × and 400 × , respectively; scale bars are 500 μm and 50 μm)

We used immunohistochemical methods to detect the expression of TMEM79 and SMG5 in HCC and adjacent normal tissues to validate the previous results. The results showed that TMEM79 and SMG5 were significantly upregulated (Fig. 5, Table 1) in HCC. Correlation analysis showed that TMEM79 and SMG5 played a role in promoting liver cancer progression (*p* = 0.001, *R* = 0.202) (Table 2).

**Table 1:** Expression of TMEM79 and SMG5 in HCC

Characteristics	*n*	TMEM79		χ2	*p* value	SMG5		χ2	*p* value
		Low or none (%)	High (%)			Low or none (%)	High (%)		
HCC	282	113 (40.1)	169 (59.9)	150.025	< 0.001^a^	99 (35.1)	183 (64.9)	91.528	< 0.001^a^
Adjacent normal	282	252 (89.4)	30 (10.6)			212 (75.2)	70 (24.8)		

*HCC* hepatocellular carcinoma

^a^*P* < 0.05 was considered significant

**Table 2:** Relationship between TMEM79 and SMG5 in HCC

		SMG5		χ2	*p* value	*R*
		Low or none	High			
TMEM79	Low or none	53 (53.5)	60 (32.8)	11.517	0.001^a^	0.202
	High	46 (46.5)	123 (67.2)			

^a^*P* < 0.05 was considered significant

### The relationships of TMEM79 and SMG5 with clinicopathological features in HCC

**Fig. 6:**
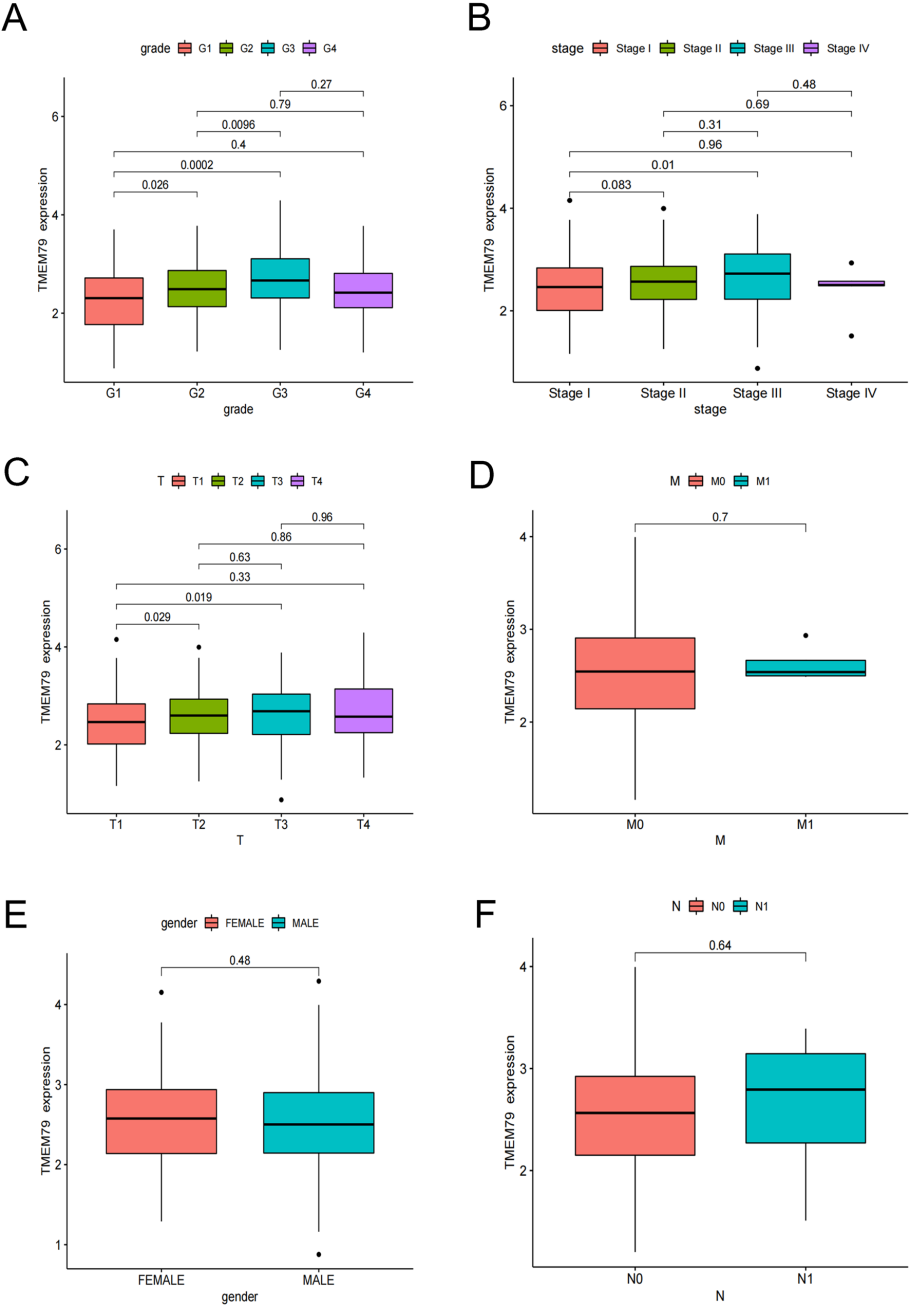
Correlations between TMEM79 and clinical characteristics of HCC. **A** Correlation of TMEM79 with grade. **B** Relationship between TMEM79 expression and TNM stage. **C** Relationship between TMEM79 expression and T-stage. **D** Correlation between TMEM79 and M-stage. **E** Relationship between TMEM79 expression and gender. **F** Relationship between TMEM79 expression and lymph node metastasis

**Fig. 7:**
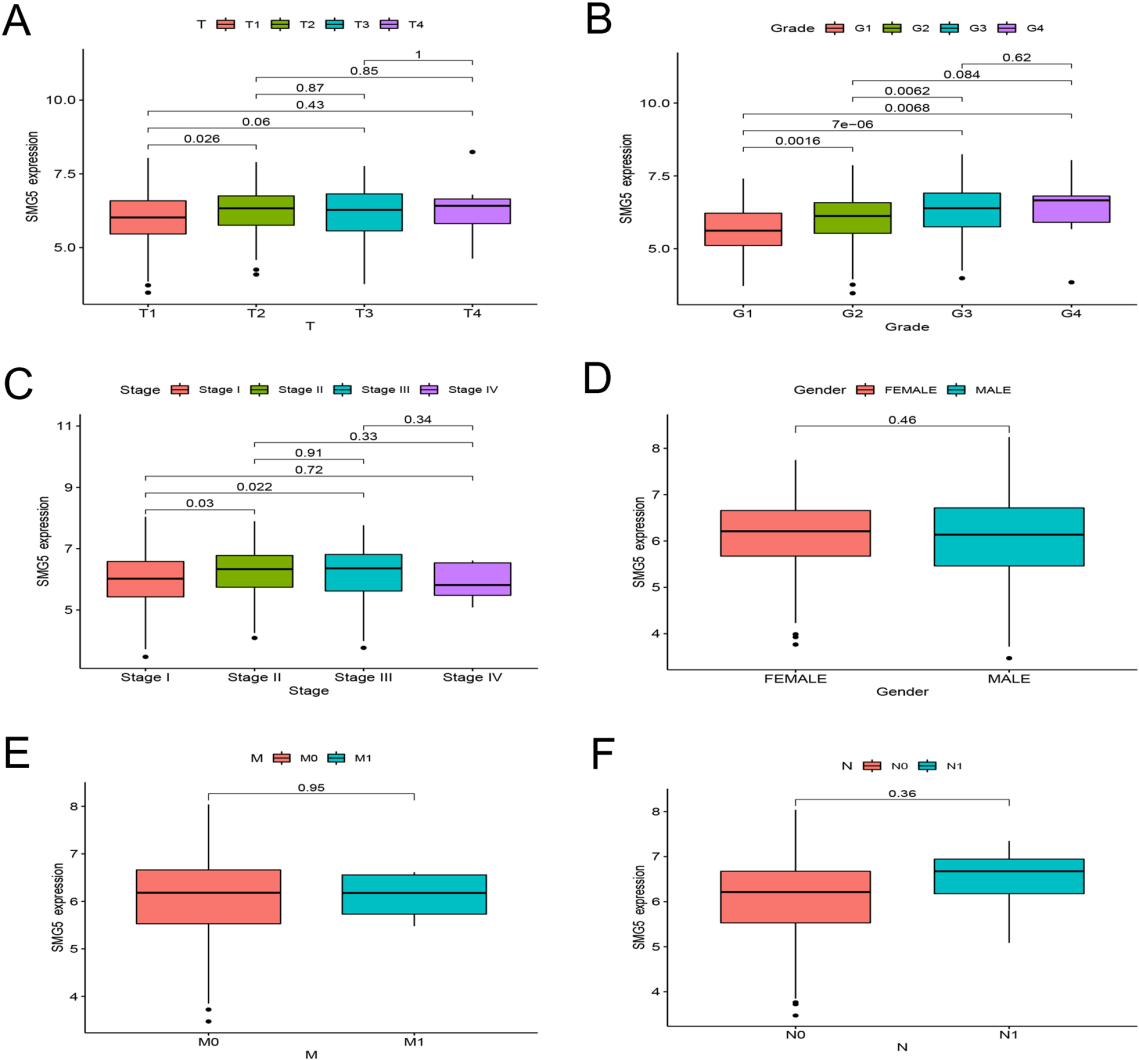
Correlations between SMG5 and clinical characteristics of HCC. **A** Correlation between SMG5 and T-stage of HCC. **B** Relationship between SMG5 expression and tumor grade. **C** Relationship between SMG5 expression and TNM staging. **D** Relationship between SMG5 expression and gender. **E** Correlation between SMG5 and M-stage. **F** Relationship between SMG5 expression and lymph node metastasis

Also we then analyzed the clinical information of the TCGA database by R software and the results showed TMEM79 was found to be higher expressed in the higher grade, higher stage and higher T grade (Fig. 6A–C). No statistically significant associations was found between TMEM79 and clinical characteristics, such as gender, and M and N stages (Fig. 6D–F). There were statistically significant differences between SMG5 and T stage, grade, and stage, with higher SMG5 expression at a higher grade (Fig. 7A–C). There were no statistical differences between SMG5 with gender, M stage, and N stage (Fig. 7D–F).

And the results in IHC showed that TMEM79 was correlated with multiple factors, such as TNM stage (*P* <  0.001), vascular invasion (*P* = 0.005), T (*P* = 0.004), M (*P* < 0.001), and recurrence (*P* = 0.004) (Table 3). SMG5 exhibited significant associations with tumor number (*P* = 0.01), T (*P* = 0.019), M (*P* = 0.002), and TNM stage (*P* = 0.001) (Table 3).

**Table 3:** TMEM79 and SMG5 expression by clinicopathological characteristics of patients with HCC

Characteristics	*n*	TMEM79 (%)		χ2	*P*	SMG5 (%)			
		Low or none	High			Low or none	High	χ2	*P*
Gender		113 (40.1)	169 (59.9)	0.016	0.507	99 (35.1)	183 (64.9)	0.095	0.432
Male	71	28 (39.4)	43 (60.6)			26 (36.6)	45 (63.4)		
Female	211	85 (40.3)	126 (59.7)			73 (34.6)	138 (65.4)		
Age (years)		113 (40.1)	169 (59.9)	0.024	0.493	99 (35.1)	183 (64.9)	3.049	0.056
< 60	211	84 (39.8)	127 (60.2)			68 (32.2)	143 (67.8)		
≥ 60	71	29 (40.8)	42 (59.2)			31 (43.7)	40 (56.3)		
Tumor diameter (cm)		113 (40.1)	169 (59.9)	1.289	0.156	99 (35.1)	183 (64.9)	0.685	0.243
< 5	176	66 (37.5)	110 (62.5)			65 (36.9)	111 (63.1)		
≥ 5	106	47 (44.3)	59 (55.7)			34 (32.1)	72 (67.9)		
AFP (μg/L)		113 (40.1)	169 (59.9)	0.025	0.492	99 (35.1)	183 (64.9)	0.291	0.345
< 400	216	86 (39.8)	130 (60.2)			74 (34.3)	142 (65.7)		
≥ 400	66	27 (40.9)	39 (59.1)			25 (37.9)	41 (62.1)		
Tumor number		113 (40.1)	169 (59.9)	2.654	0.071	99 (35.1)	183 (64.9)	5.848	0.010^a^
Solitary	243	102 (42.0)	141 (58.0)			92 (37.9)	151 (62.1)		
Multiple	39	11 (28.2)	28 (71.8)			7 (17.9)	32 (82.1)		
TNM stage		113 (40.1)	169 (59.9)	22.021	< 0.001^a^	99 (35.1)	183 (64.9)	15.918	0.001^a^
I	193	91 (47.2)	102 (52.8)			77 (39.9)	116 (60.1)		
II	53	20 (37.7)	33 (62.3)			20 (37.7)	33 (62.3)		
III	15	1 (6.7)	14 (93.3)			1 (6.7)	14 (93.3)		
IV	21	1 (4.8)	20 (95.2)			1 (4.8)	20 (95.2)		
T		113 (40.1)	169 (59.9)	13.464	0.004^a^	99 (35.1)	183 (64.9)	9.918	0.019^a^
1	193	90 (46.6)	103 (53.4)			77 (39.9)	116 (60.1)		
2	71	21 (29.6)	50 (70.4)			21 (29.6)	50 (70.4)		
3	12	2 (16.7)	10 (83.3)			1 (8.3)	11 (91.7)		
4	6	0 (0.0)	6 (100.0)			0 (0.0)	6 (100.0)		
N		113 (40.1)	169 (59.9)	0.395	0.417	99 (35.1)	183 (64.9)	0.135	0.529
0	275	111 (40.4)	164 (59.6)			97 (35.3)	178 (64.7)		
1	7	2 (28.6)	5 (71.4)			2 (28.6)	5 (71.4)		
M		113 (40.1)	169 (59.9)	11.026	< 0.001^a^	99 (35.1)	183 (64.9)	8.565	0.002^a^
0	262	112 (42.7)	150 (57.3)			98 (37.4)	164 (62.6)		
1	20	1 (5.0)	19 (95.0)			1 (5.0)	19 (95.0)		
Differentiation		113 (40.1)	169 (59.9)	1.944	0.378	99 (35.1)	183 (64.9)	4.949	0.084
Well	33	11 (33.3)	22 (66.7)			17 (51.5)	16 (48.5)		
Moderate	159	61 (38.4)	98 (61.6)			55 (34.6)	104 (65.4)		
Poor	90	41 (45.6)	49 (54.4)			27 (30.0)	63 (70.0)		
Hepatitis B virus infection		113 (40.1)	169 (59.9)	1.210	0.199	99 (35.1)	183 (64.9)	1.872	0.134
No	18	5 (27.8)	13 (72.2)			9 (50.0)	9 (50.0)		
Yes	264	108 (40.9)	156 (59.1)			90 (34.1)	174 (65.9)		
Vascular invasion		113 (40.1)	169 (59.9)	7.094	0.005^a^	99 (35.1)	183 (64.9)	0.381	0.328
No	231	101 (43.7)	130 (56.3)			83 (35.9)	148 (64.1)		
Yes	51	12 (23.5)	39 (76.5)			16 (31.4)	35 (68.6)		
Liver cirrhosis		113 (40.1)	169 (59.9)	2.202	0.087	99 (35.1)	183 (64.9)	0.950	0.198
No	137	61 (44.5)	76 (55.5)			52 (38.0)	85 (62.0)		
Yes	145	52 (35.9)	93 (64.1)			47 (32.4)	98 (67.6)		
Recurrence		113 (40.1)	169 (59.9)	7.667	0.004^a^	99 (35.1)	183 (64.9)	0.526	0.289
No	230	101 (43.9)	129 (56.1)			83 (36.1)	147 (63.9)		
Yes	52	12 (23.1)	40 (76.1)			16 (30.8)	36 (69.2)		

^a^*P* < 0.05 was considered significant

### Columnar plots for predicting prognosis of HCC patients in TCGA database

**Fig. 8:**
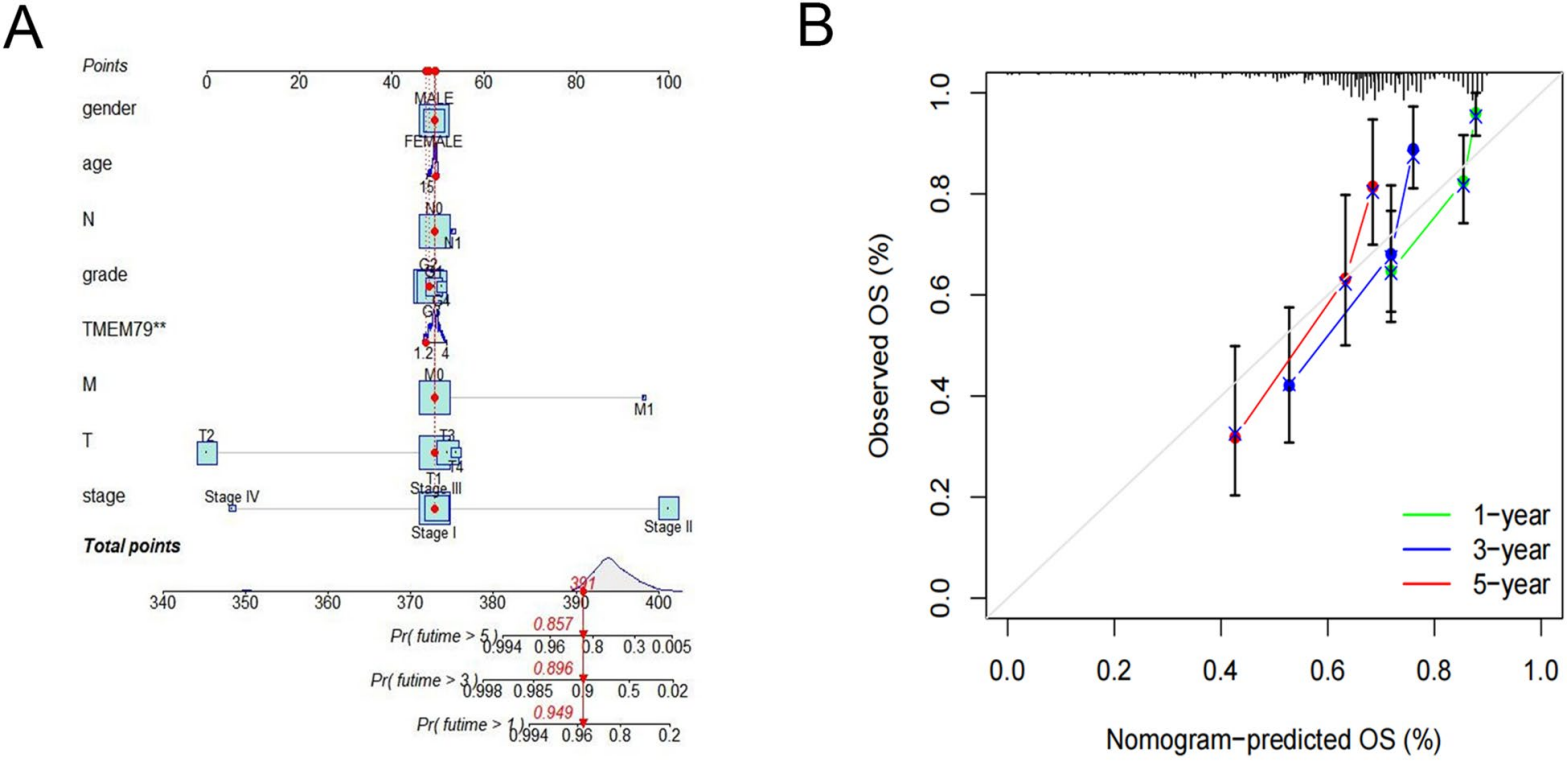
Predicting prognosis of HCC patients using nomogram in TCGA database. A Construction and validation of the prognostic column line graph of HCC. B Columnar line plot calibration curves for 1-year, 3-year, and 5-year OS prediction

Based on the TCGA database, columnar plots were established to predict the 1-year, 3-year, and 5-year OS for patients with HCC. The parameters used were the final expression level of TMEM79, gender, age, T-stage, N-stage, grade, M stage, and clinical stage. The ideal model fits better with calibration curves for OS at 1, 3, and 5 years (Fig. 8A, B).

### Correlations of TMEM79 and SMG5 with prognosis of HCC patients in our research

**Fig. 9:**
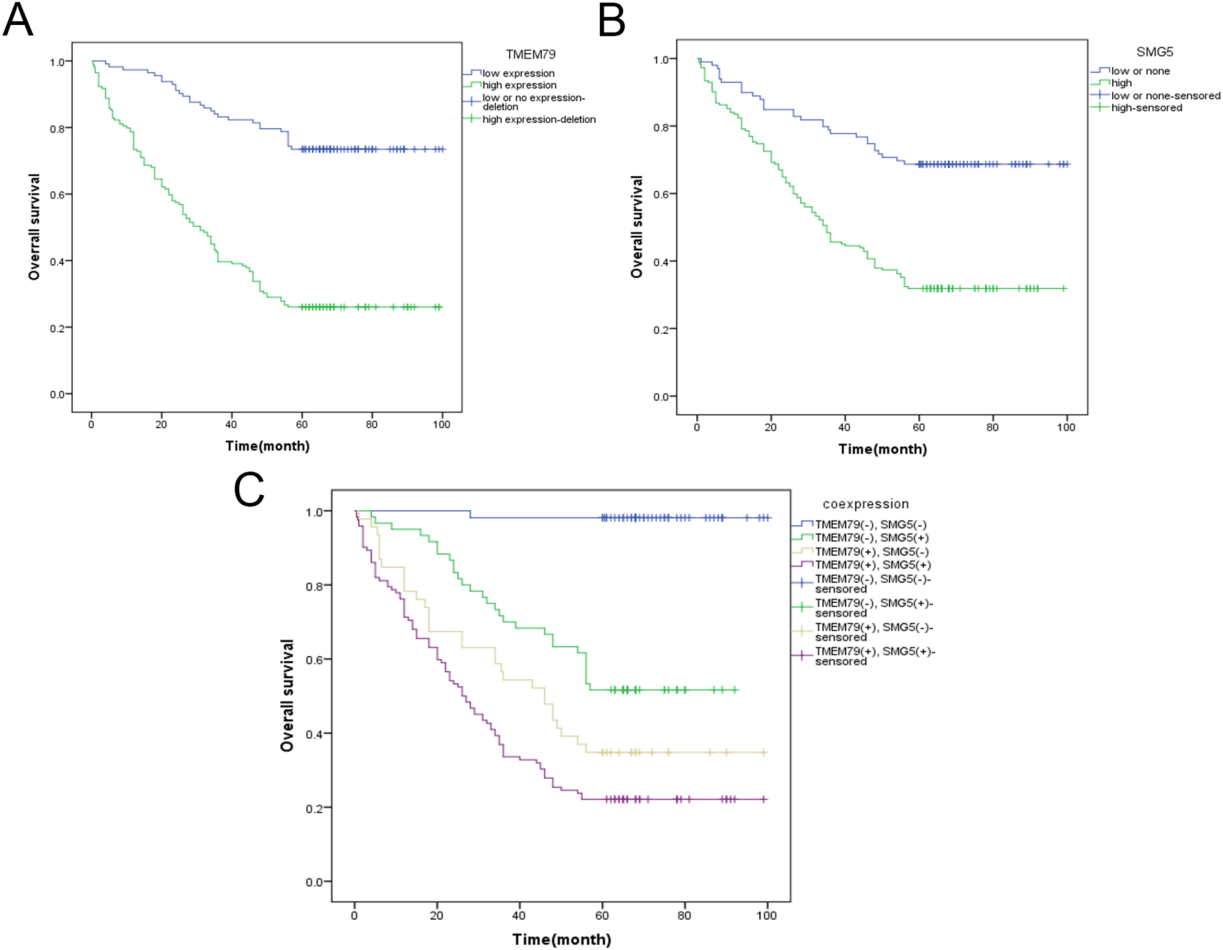
Kaplan–Meier curves of TMEM79 and SMG5 in HCC in our research. **A** Kaplan–Meier curve of TMEM79 (*P* < 0.001). **B** Kaplan–Meier curve of SMG5 (*P* < 0.001). **C** Kaplan–Meier curve of co-expression of TMEM79 and SMG5 (*P* < 0.001)

The Kaplan–Meier analysis in IHC showed that high-expression TMEM79(*X*^2^ = 66.549, *P* < 0.001) and SMG5(*X*^2^ = 32.117, *P* < 0.001) revealed poorer prognosis of HCC patients (Fig. 9A, B). Patients with HCC have poorer prognosis when TMEM79 and SMG5 were both expressed at higher levels (Fig. 9C) (*X*^2^ = 84.225, *P* < 0.001). Further univariate and multivariate analyses demonstrated that TMEM79 (HR = 4.552, *P* < 0.001) and SMG5 (HR = 2.945, *P* < 0.001) were independent prognostic factors in HCC (Table 4).

**Table 4:** Univariate and multivariable analyses of survival factors in patients with HCC

	Univariate analysis				Multivariate analysis			
	HR	*P* >|z|	95% CI		HR	*P* >|z|	95% CI	
TMEM79 expression								
High vs low or none	4.552	< 0.001^a^	3.047	6.801	3.833	< 0.001^a^	2.520	5.831
SMG5 expression								
High vs low or none	2.945	< 0.001^a^	1.984	4.372	1.903	0.002^a^	1.257	2.883
Age (years)								
≤ 60 vs > 60	1.001	0.995	0.694	1.444				
Gender								
Male vs female	1.183	0.373	0.818	1.712				
Tumor diameter (cm)								
≤ 3 vs > 3	1.482	0.015^a^	1.079	2.037	1.446	0.039^a^	1.019	2.051
AFP (μg/L)								
≤ 400 vs > 400	1.091	0.644	0.754	1.579				
Tumor number								
Solitary vs multiple	0.491	0.001^a^	0.328	0.737	0.976	0.927	0.578	1.647
TNM stage								
I vs II vs III + IV	1.878	< 0.001^a^	1.612	2.187	1.487	0.119	0.904	2.446
T								
1 vs 2 vs 3 vs 4	2.274	< 0.001^a^	1.839	2.813	1.338	0.179	0.875	2.044
N								
0 vs 1	2.005	0.126	0.822	4.895				
M								
0 vs 1	4.278	< 0.001^a^	2.590	7.066	0.910	0.852	0.338	2.451
Differentiation								
Well vs moderate vs poor	1.211	0.146	0.936	1.566				
Hepatitis B virus infection								
No vs yes	0.762	0.384	0.412	1.406				
Vascular invasion								
No vs yes	1.763	0.003^a^	1.209	2.571	0.351	0.318	0.045	2.734
Liver Cirrhosis								
No vs yes	0.945	0.723	0.689	1.294				
Recurrence								
No vs yes	1.812	0.002^a^	1.247	2.633	1.968	0.508	0.265	14.638

*HCC* hepatocellular carcinoma, *HR* hazard ratio, *CI* confidence interval

^a^*P* < 0.05 was considered significant

### Enrichment analysis of TMEM79 in the TCGA database

**Fig. 10:**
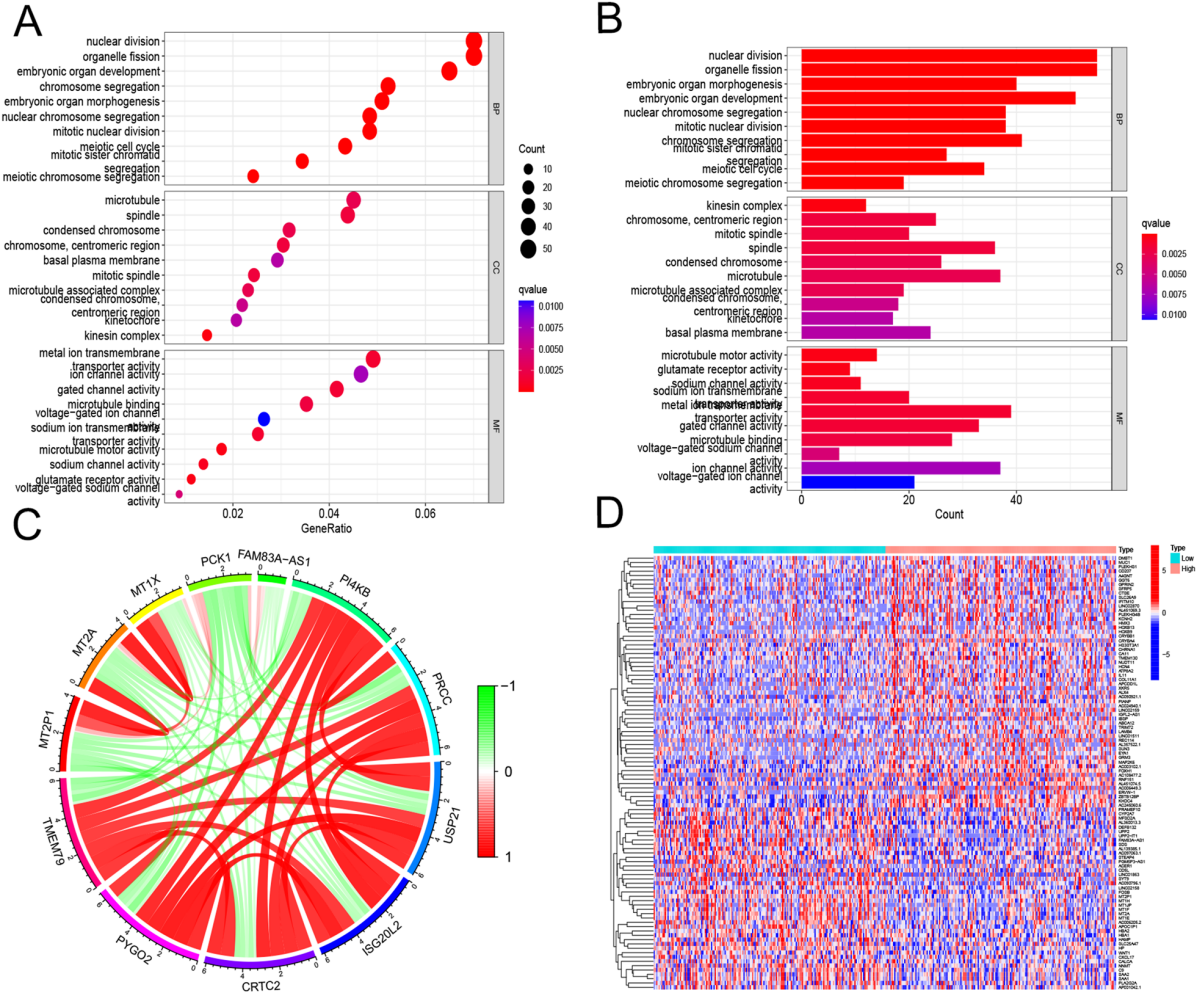
GSEA analysis results based on TCGA database. **A**, **B** Enrichment analysis of TMEM79 by GO and KEGG. **C** Circos plot of the co-expression network of TMEM79 with 11 genes in HCC samples. **D** Heatmap showed down-regulated genes (blue) and up-regulated genes (red) identified in the high expression group

Based on the RNA sequencing data from the TCGA database, TMEM79-related genes were found to be mainly enriched in the nuclear division, mitosis, embryonic organ development, nuclear chromosome segregation, meiosis, microtubule microfilaments, and transport channel proteins. Our filtering conditions are | log FC |> 1 and FDR < 0.05. In addition, GSEA analysis showed that immune-related cellular functions were enriched in HCC patients with low TMEM79 expression (Fig. 10A, B). Circos showed co-expression network of TMEM79 and 11 genes (Fig. 10C). Heatmap showed down-regulated genes (blue) and up-regulated genes (red) identified in the high expression group (Fig. 10D).

### Correlations of TMEM79 and SMG5 with immune cell infiltration in HCC in the TCGA database

**Fig. 11:**
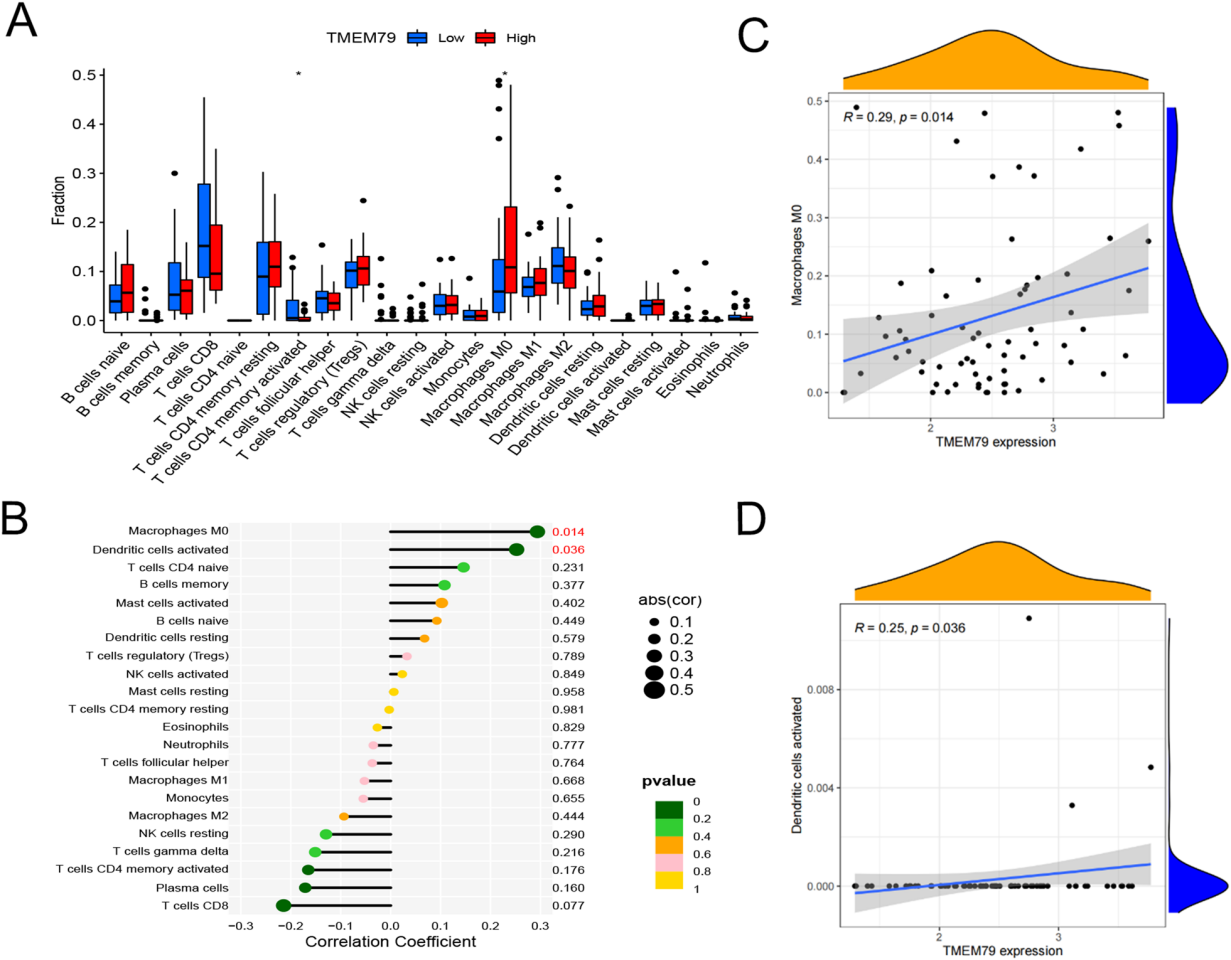
Correlation of TMEM79 with immune cell infiltration in HCC. **A** comparison of immune cell infiltration in the TMEM79 high expression group and low expression group. **B** Lollipop shows the correlation between immune infiltration and TMEM79. **C**, **D** Correlation of immune infiltration with TMEM79 expression

**Fig. 12:**
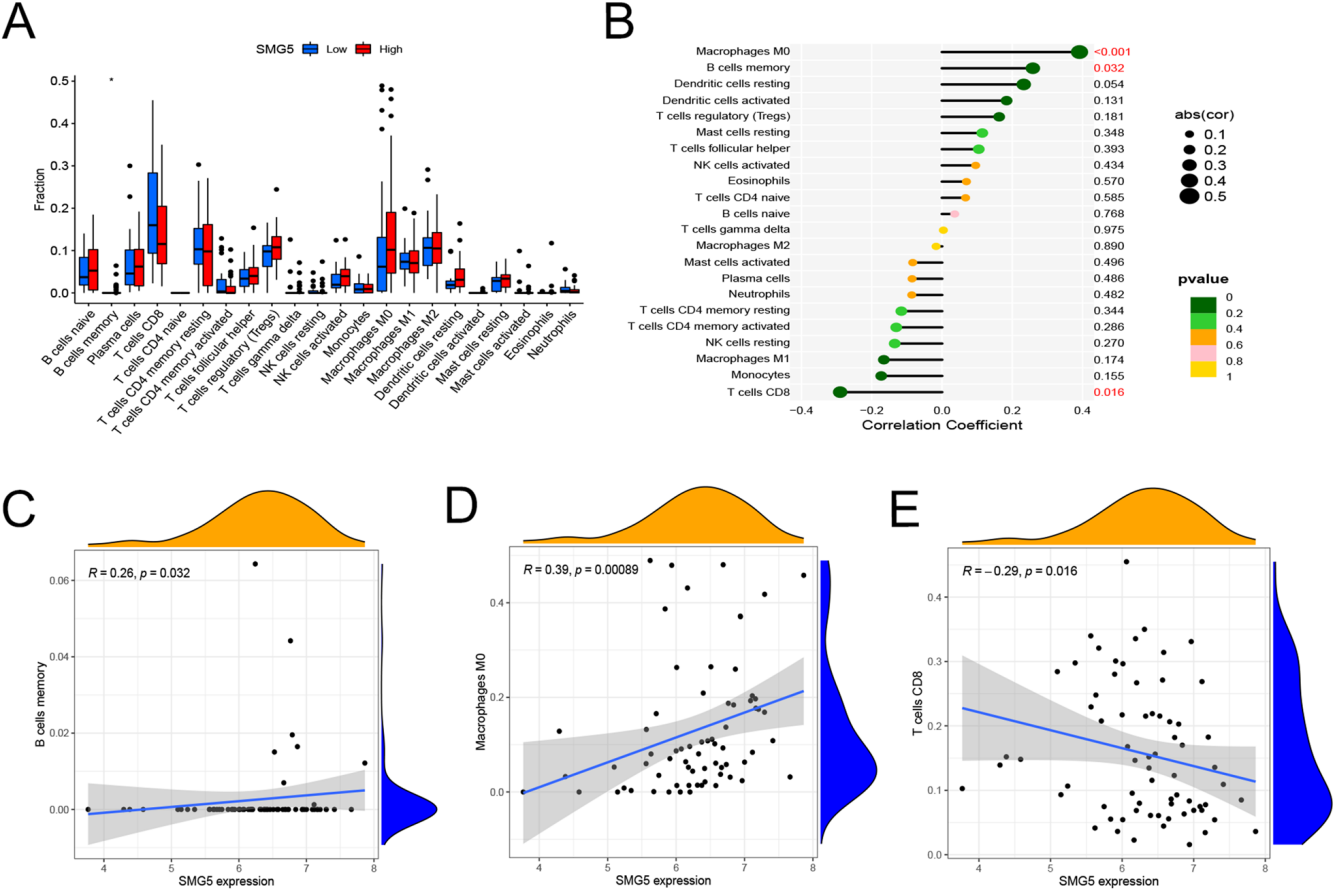
Correlation of SMG5 with immune cell infiltration in HCC. **A** Comparison of immune cell infiltration in the SMG5 high expression group and low expression group. **B** Lollipop shows the correlation between immune infiltration and SMG5. **C**, **D**, **E** Correlation between SMG5 and B cells, M0 macrophages and CD8 + T cells

Differential analysis of immune cell infiltration levels between high and low TMEM79 expression groups was performed based on ‘CIBERSORT’. TMEM79 showed a noteworthy correlation with the immune cells that infiltrated the tumor. Macrophage and dendritic cell infiltration levels were elevated in the high TMEM79 expression group (Fig. 11A). Macrophage and dendritic cell infiltration levels were positively correlated with TMEM79 (Fig. 11B–D). According to TIMER (https://cistrome.shinyapps.io/timer/), TMEM79 expression levels were positively correlated with CD4 + T cells, macrophages, bone-marrow dendritic cells, and neutrophils. In the same way, the analysis of immune cell infiltration was performed for SMG5.High expression of SMG5 was related to B cells, M0 macrophages, and CD8 + T cells (Fig. 12A). Lollipop showed the correlation between immune infiltration and SMG5 (Fig. 12B). The results showed that SMG5 expression was positively correlated with the level of B-cell and M0 macrophage infiltration and negatively correlated with CD8-positive T-cell immune cell infiltration (Fig. 12C–E).

### Associations of TMEM79 and SMG5 with immune checkpoints and drug responsiveness in HCC in TCGA database

**Fig. 13:**
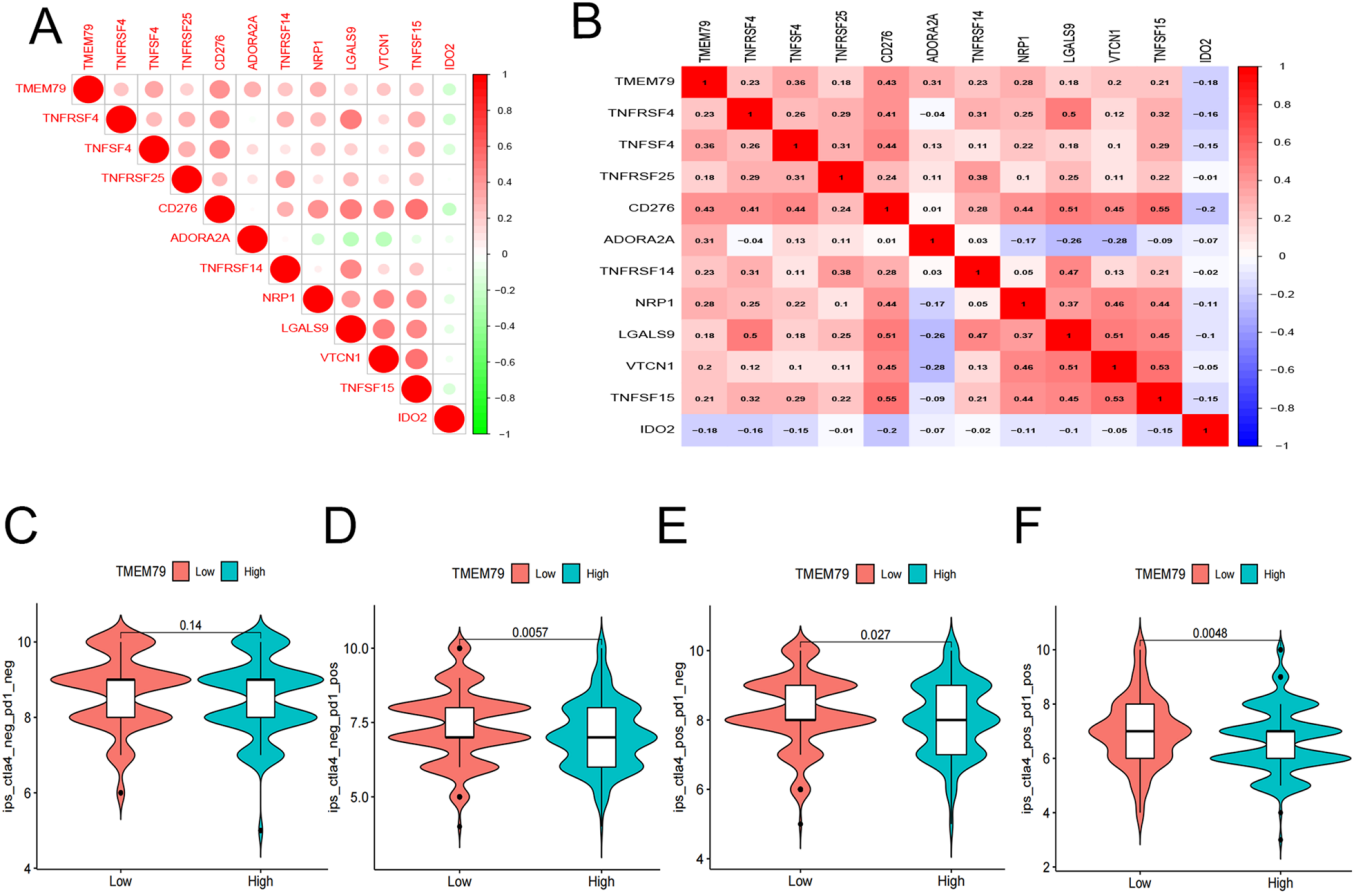
Role of TMEM79 on immune checkpoints and drug sensitivity. **A**, **B** Correlation of TMEM79 with immune checkpoints. **C** Correlation of expression of TMEM79 with CTLA4-negative and PD1-negative efficacy. **D** Correlation of expression of TMEM79 with CTLA4-negative and PD1-positive efficacy. **E** Correlation of expression of TMEM79 with CTLA4-positive and PD1-negative efficacy. **F** Correlation of expression of TMEM79 with CTLA4-positive and PD1-positive efficacy

**Fig. 14:**
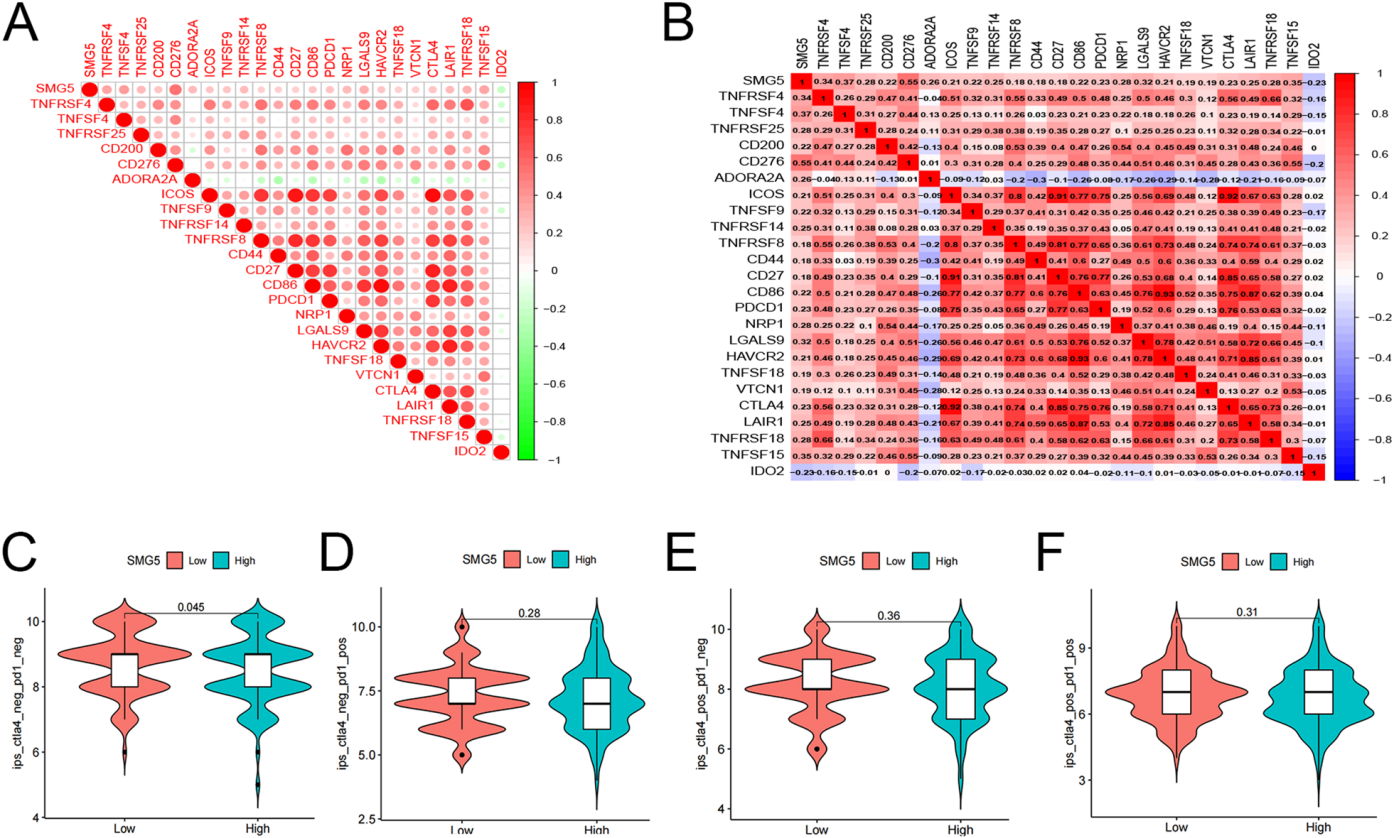
Role of SMG5 on immune checkpoints and drug sensitivity. **A**, **B** Correlation of SMG5 with immune checkpoints. **C** Correlation of expression of SMG5 with CTLA4-negative and PD1-negative efficacy. **D** Correlation of expression of SMG5 with CTLA4-negative and PD1-positive efficacy. **E** Correlation of expression of SMG5 with CTLA4-positive and PD1-negative efficacy. **F** Correlation of expression of SMG5 with CTLA4-positive and PD1-positive efficacy

Initially, we investigated the association between TMEM79 and immune checkpoint inhibitors by analyzing the expression levels of 23 immune checkpoint-related genes along with TMEM79. TMEM79 was significantly correlated with all 11 immune checkpoint-related genes (*P* < 0.05) [13]. Among them, TMEM79 was negatively correlated with IDO2 and positively correlated with LGALS9, NRP1, CD276, VTCN1, TNFSF4 (Fig. 13A, B). Anti-CTLA4 and anti-PD1 inhibitors also had higher treatment scores, prediting better treatment outcomes and better drug sensitivity. Statistical significance was found only in CTLA4-positive PD1-positive sections, suggesting that the expression of TMEM79 was associated with immunotherapy (anti-CTLA4 and anti-PD1 therapy) and combined use of both was a better option for patients with low TMEM79 expression (Fig. 13C–F). Similarly, SMG5 is associated with 24 immune checkpoints, SMG5 is positively correlated with immune checkpoint action, and has the most pronounced effect with CD276 (Fig. 14A, B). There was no statistically significant difference between SMG5 and immunotherapy (anti-CTLA4 and anti-PD1 therapy) (Fig. 14C–F).

### TMEM79-related molecular in HCC in databases

**Fig. 15:**
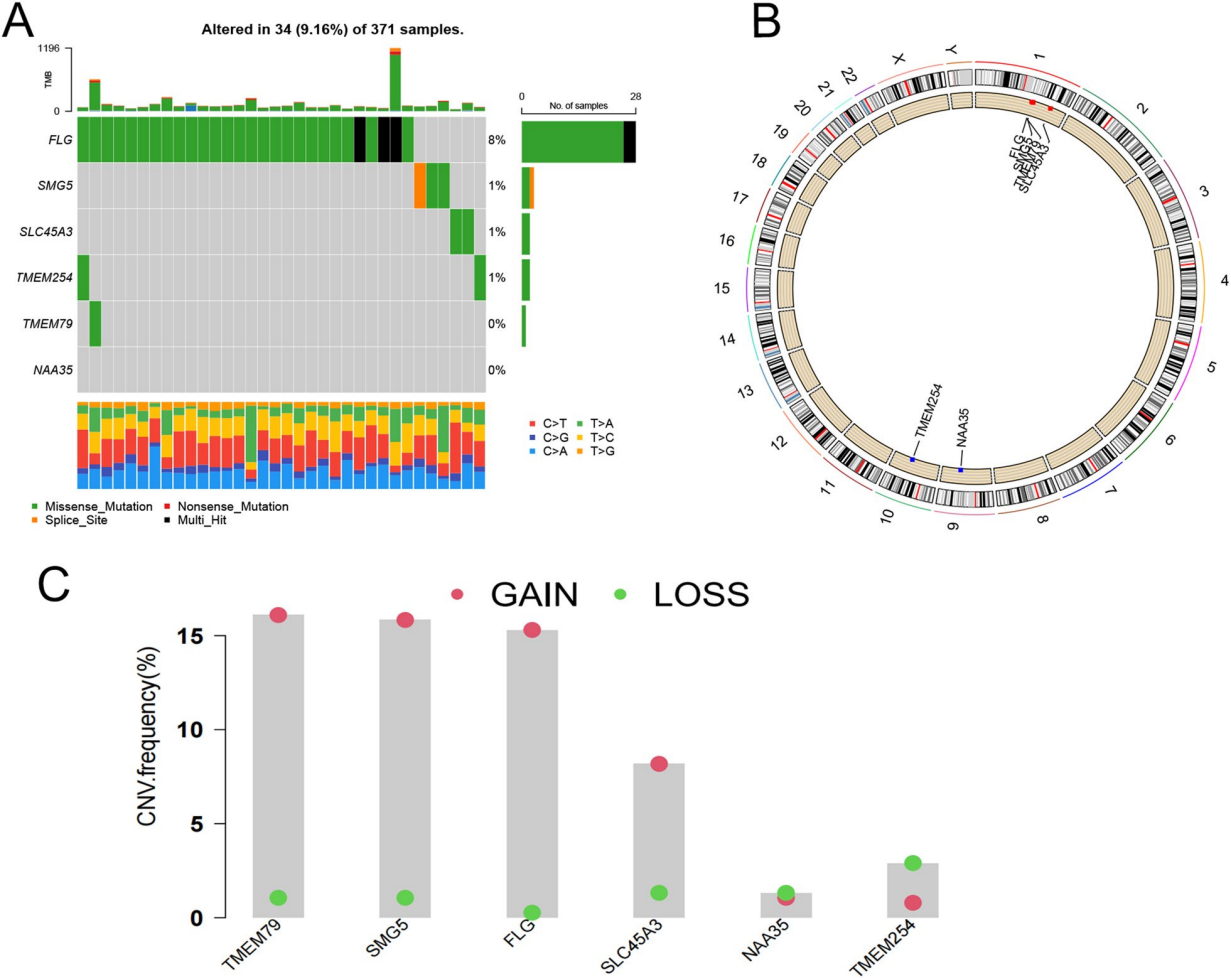
Analysis of genes associated with TMEM79. **A** Data on mutation frequency in patients with HCC. **B** CNV variant loci of associated genes on chromosomes. **C** Distribution of CNV gains, losses, and non-CNVs in associated genes

String online analysis of TMEM79-associated reciprocal proteins was performed to further analyze these 10 genes. The expression levels of TMEM79-related derivatives in 374 liver cancer patients and 50 normal human liver tissues were examined on the TCGA data sets. In addition, mutation analysis showed that 34 of 371 samples (9.16%) had mutations, with FLG having the highest mutation rate. Based on pairwise copy number changes and all 6 genes had frequent copy number changes (Fig. 15A–C).

### Identification of different subtypes in HCC in databases

**Fig. 16:**
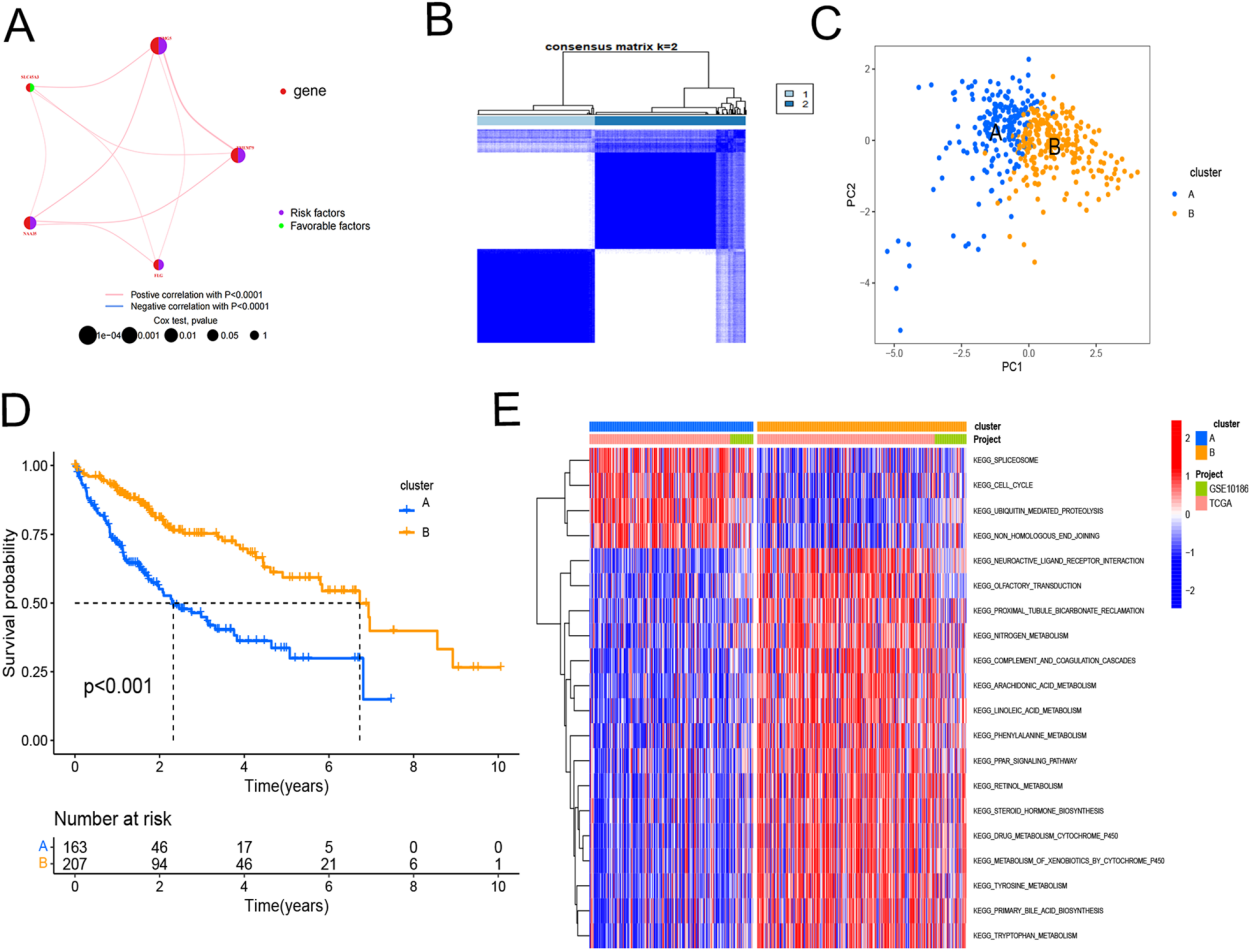
Clinical phenotypic analysis of TMEM79-related molecular typing. **A** Biological and clinicopathological characterization of the factors of interest. **B** Consensus matrix heat map of 2 clusters (κ = 2). **C** Presence of transcriptomic differences identified by PCA analysis. **D** K–M curves of subtypes A and subtypes B. **E** For subtypes A and subtypes **B** related pathways in HCC (red and blue strings indicate positive and negative correlations, respectively; strength of correlations is indicated by hue)

A correlation network plot illustrates a robust correlation among 5 genes (Fig. 16A). We next performed consensus clustering analysis to classify HCC samples into different patterns, and two distinct clusters were identified using unsupervised clustering (Fig. 16B). We named these clusters A–B and principal component analysis of cluster showed obvious segregation among two clusters (Fig. 16C). According to Kaplan–Meier survival calculations, subtype B had a better prognosis than subtype A (*P* < 0.001; Fig. 16D). By KEGG, these clusters were correlated with CELLCYCLE, SPLICEOSOME, UBIQUITINMEDIATEDPROTEOLYSIS and so on (Fig. 16E).

### TME infiltration and functional enrichment of different subtypes in the TCGA database

**Fig. 17:**
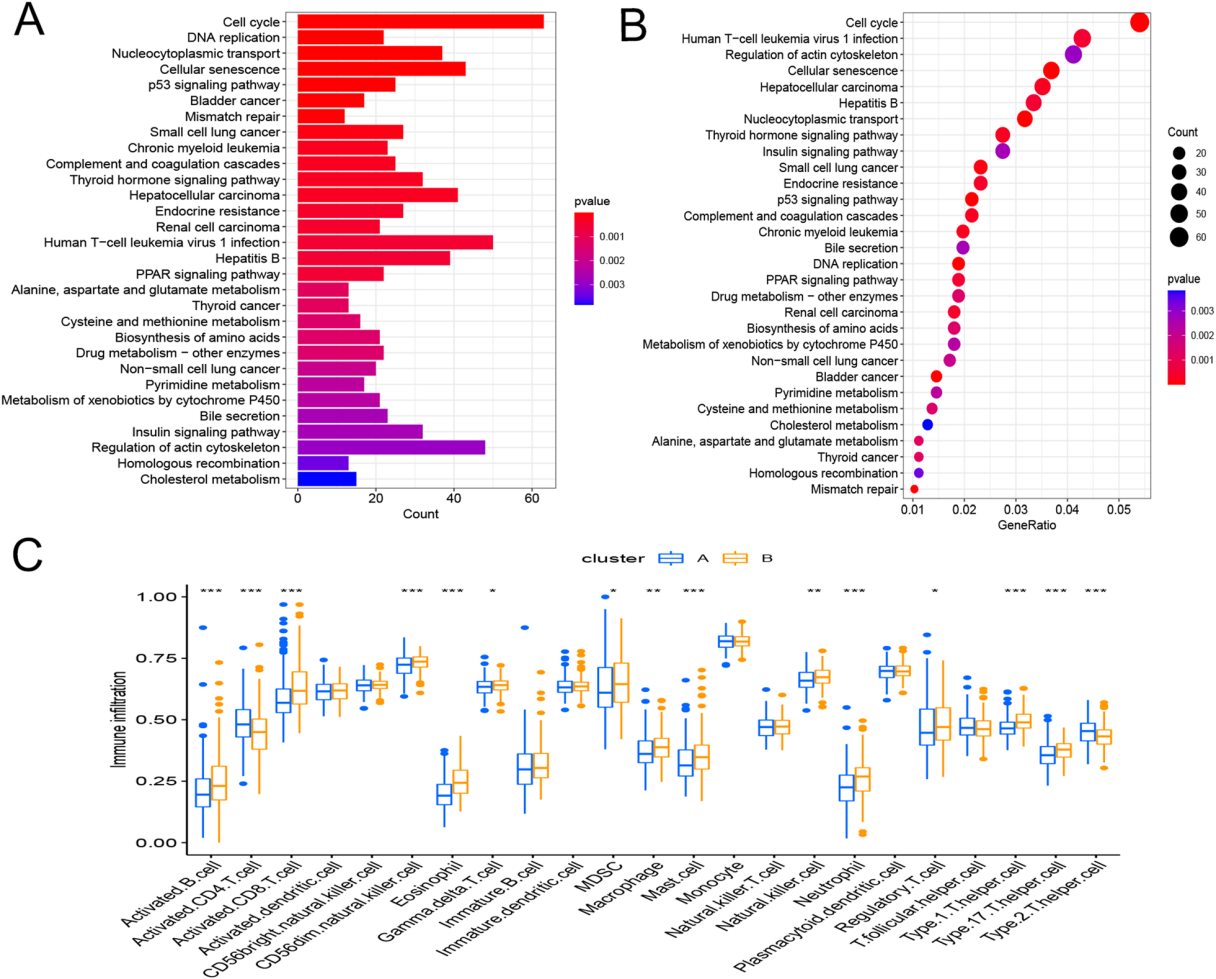
Pathway enrichment analysis of tumor typing of TMEM79-related molecules. **A**, **B** Enrichment analysis of different subtypes using GO and KEGG. **C** Correlation between the levels of immune cell infiltration in the relevant subtypes. *P < 0.05, **P < 0.01, ***P < 0.001

We used gene-set variant analysis enrichment analysis to investigate the possible effects of both isoforms on biological behavior. Subtype A was found to be enriched in pathways related to immune activation when compared to subtype B. According to GSVA enrichment studies, isoform A is considerably enriched in metabolic activation pathways, such as folate utilization of a carbon pool, lysine degradation, citrate cycle, RNA metabolism, arachidonic acid metabolism and N-glycan biosynthesis (Fig. 17A, B). In each HCC sample, we used the CIBERSORT approach to assess the association between the two isoforms and 23 other immune cell isoforms to learn more about how they work in the tumor microenvironment. According to our study, the number of immune cells infiltrated by both subtypes in CD4 T cells, type 2 T helper cells, regulatory T cells, γ δ T cells, immature dendritic cells and immature B cells were found to be more prevalent in subtype A compared to subtype B (Fig. 17C).

## Discussion

TME is a complex and rich multicellular environment for tumor growth [14]. Tumor-associated immune cells including macrophages, T cells, B cells, natural killer cells, and tumor-associated neutrophils are involved in tumor immune responses, affecting the TME and regulating tumor growth and metastasis [15]. The main body immune system responsible for supervising and killing tumor cells are killer NK cells, and the cells are effector lymphocytes with the ability to generate anti-tumor responses [16]. Targeted therapy of TME is considered an important method for treating tumors. At present, the clinical application of drugs and cell therapies for immune checkpoints and T cells have driven further exploration of TME to find other new targets that can be used. At present, there are several immune checkpoints, including PD-1, CTLA-4, LAG-3, and TIGIT, which have both distinct and shared inhibitory roles in regulating the activation. differentiation, and function of T cells [17]. Therefore, the search for more immune checkpoints is one of the directions to treat tumors.

We downloaded transcriptome and clinical data of liver cancer from the TCGA database, and then screened eligible molecules using R software. We found that TMEM79 was differentially expressed in liver cancer tissues and normal tissues. TMEM79 is a member of the transmembrane protein (TMP) family that encodes for transmembrane protein79. TMPs play a significant function in cells, serving as transporter proteins and receptors [18]. In addition, TMP plays a crucial role in cells that act primarily as transporter proteins and receptors. TMEM79 (transmembrane protein 79), a protein-coding gene that helps maintain epidermal integrity and skin barrier function, plays a role in the formation of the lamellar granule (LG) secretion system and the stratum corneum (SC) epithelium [19]. TMEM79 may be involved in multiple processes, including epithelial cell maturation, establishment of the skin barrier, and positive regulation of epidermal development. TMEM79 acts mainly upstream or within the keratinization which affects the development of the stratum corneum and the morphogenesis of the hair follicle. TMEM79 can interact with ubiquitin-specific protease 8 (USP8) [20], leading to human tumorigenesis. TMEM79 is a potential novel biomarker for BPH [6], and may act as a pivotal factor involved in immune response and tumor cell development in malignant melanoma tumorigenesis [21].

Transcription induced chimeric RNAs possess sequences from different genes, and are expected to increase proteomic diversity through chimeric proteins or altered regulation. The prevalence of chimeric RNA may allow a limited number of human genes to encode large amounts of RNA and proteins, forming an additional layer of cellular complexity. According to reports, TMEM79–SMG5 is present in tumor tissue. It can be used to distinguish between tumor patients and non-tumor patients. Therefore, it is most likely to become a diagnostic marker for tumors [22]. Then, we discovered the interaction between TMEM79 and SMG5 by String database. The discovery of SMG5 may be related to the prognosis of HCC. Therefore, we chose them for research in HCC tissue TMEM79–SMG5 is highly expressed in prostate cancer cells. Through previous studies, we found that TMEM79 may play a potential role in promoting HCC, and SMG5 may play a common role in promoting the development of HCC. SMG5 may be associated with memory B cells, M0 macrophages, neutrophils, activated memory CD4 + T cells, follicular helper T cells and regulatory T cells in HCC [10]. SMG5 can be used to predict the prognosis of HCC in the current study [23] and may be associated with sex- and race-specific prognostic variability in gastric cancer [11]. SMG5 is involved in nonsense-mediated mRNA decay [24] and enhances the dephosphorylation of UPF1[24]. SMG5 is thought to provide a link to mRNA degradation mechanisms involving the extra nucleoside catabolic pathway and acts as an adapter of UPF1 to protein phosphatase 2A (PP2A), thereby triggering UPF1 dephosphorylation [24].

Targeted therapy of the TME has been considered a very promising anti-cancer strategy [14]. The clinical approval of drugs targeting the vascular system, immune checkpoint inhibitors, and T-cell therapy have benefited many patients [14]. However, because of the complexity and variability of the TME, a single target may not be sufficient to control tumor progression, and the combination of multiple approaches can exert better therapeutic effects [25]. As a result, we must identify additional therapeutic targets. Based on the previously known results, SMG5 plays an important role in HCC. Therefore, we further investigated through what pathway SMG5 affects HCC and whether it could be a potential immune checkpoint for HCC.

In this present study, we analyzed the expression of TMEM79 and SMG5 in HCC. The results showed that both TMEM79 and SMG5 were highly expressed in HCC. Prognostic analysis of TMEM79 and SMG5 suggested that they could act as independent prognostic factors in HCC and affect the prognosis of patients with HCC. Based on the results of TCGA database, we went to verify the expression and prognostic role of both in patients with HCC in our data sample. The results showed that the expressions of TMEM79 and SMG5 were higher in HCC than in adjacent tissues. Patients with TMEM79 and SMG5 high expression of HCC had poor OS. Patients with high TMEM79 and SMG5 expression had higher tumor stage and were more likely to metastasize to distant sites.

Next, we explored the pathways by which the expressions of TMEM79 and SMG5 may affect the prognosis of patients with HCC. TMEM79 were found to be mainly enriched in the nuclear division, mitosis, embryonic organ development, nuclear chromosome segregation, meiosis, microtubule microfilaments, and transport channel proteins. There was a significant correlation between the expression of TMEM79 and tumor-infiltrating immune cells, and a positive correlation between macrophages and dendritic cells. The expression of SMG5 was positively correlated with the level of B-cell and M0 macrophage and negatively correlated with CD8-positive T-cell immune cell infiltration. Expressions of TMEM79 and SMG5 in HCC patients were positively correlated with some immune checkpoints. Drug sensitivity analysis showed a negative correlation between the expression of TMEM79 and nevirapine, angiogenesis inhibitors and sunitinib. Based on protein interaction analysis, we identified several major factors that interacted, namely, SLC45A3, NAA35, SMG5, SFTPC, FLG, TNMD, CLEC7A, FNDC4, TMEM254, and VSTM2A. For further study, we further analyzed the TMEM79-related derivatives. SMG5 has been reported to be associated with the prognosis of patients with HCC [10]. SMG5 may be associated with the OS of HCC patients. It could represent a potential drug target and help to optimize future clinical treatment [26].

Potential clinical application of GOLM1–NAA35 chimeric RNA (seG–NchiRNA) in esophageal squamous cell carcinoma (ESCC) [27]. The remaining molecules were not found to be correlated with tumor progression in known studies at this time. Our study identified two distinct molecular isoforms in HCC. Patients with subtype A had more severe clinical features and shorter OS compared to subtype B. Individuals with high expression of NAA35, SMG5, and TMEM79 had a poor prognosis. The effect of gene expression patterns on overall survival in HCC was also investigated. In addition, we compared the characteristics of the two TME subtypes and the changes in immune-related biochemical pathways. Immune activation in the HCC subtype was also largely due to the activation of B cells, CD4 T cells, CD8 T cells, regulatory T cells, mast cells, neutrophils, type 2 T helper cells, and CD56 attenuated natural killer cells. The tumor microenvironment consists of tumor cells and their surrounding cells, such as lymphocytes, tumor-infiltrating immune cells, and tumor vascular system [28]. The above two subtypes are closely related to the abundance of immune cell infiltration in HCC and provide new ideas for tumor immunotherapy and immune infiltration.

## Conclusion

TMEM79 is highly expressed in HCC and positively correlated with SMG5, which together affect the development of HCC and the survival prognosis of patients. The occurrence of this process may be related to the effects of both on the tumor microenvironment, including the level of immune cell infiltration and immunization checkpoints and drug sensitivity. Notably, TMEM79 may serve as a potential tumor immune marker for immunotherapy in HCC.

## Methods

### Clinical samples in our experiment

We used 282 pairs of HCC tissue clinical specimens tissues from department of Pathology, Affiliated Hospital of Nantong University from January 2013 to December 2019 for immunohistochemical analysis. All patients were diagnosed with primary HCC and did not receive any treatment before surgery. The clinicopathologic features of the HCC specimens were confirmed by two experienced pathologists according to the eighth-edition TNM classification of tumors. The period from the diagnosis until death (from HCC only) was defined as overall survival (OS). The longest follow-up period was 100 months, and the death toll is 127. This study was approved by the Ethics Committee of Affiliated Hospital of Nantong University and all patients had written informed consent.

### Patient information in databases

The TCGA database provided RNA sequencing information as well as clinical data for the corresponding patients. Clinical characteristics of HCC patients included age, family history, ethnicity, new tumor events, radiation therapy records, history of neoadjuvant therapy, clinical stage, tumor (T), lymph nodes (N), metastasis (M), and gender. Survival data of liver cancer patients was downloaded from the GEO database (GSE10186) (https://www.ncbi.nlm.nih.gov/geo/).

### Gene expression differences analysis

Using R4.2.1 software, we have identified differential expression of TMEM79 in HCC and adjacent normal tissues, we examined the levels of TMEM79 and SMG5 expression in 374 primary HCC tissues and 50 adjacent normal liver tissues taken from patients with varying tumor grades and stages in the TCGA database. We explored the pan-cancer expression levels of TMEM79, and SMG5 between normal and tumor samples using TIMER.

### Correlation analysis

We used the String database (https://string-db.org) to analyze interacting proteins of TMEM79 online. We studied the correlation of TMEM79 with other molecules through the online site cBioPortal.

### K–M survival curve and prognostic analysis

In the TCGA database, 424 HCC patients were categorized into groups based on their expression levels of TMEM79 and SMG5, with a distinction made between those with high and low levels. The optimal cutoff value was determined based on all possible cutoff values between the lower and upper quartiles. Patients diagnosed with HCC were separated into two groups, one with high expression and the other with low expression, according to the median cutoff values. Survival analysis was performed using R4.2.1 to construct K–M survival curves for both groups of patients to assess whether TMEM79 and SMG5 were prognostic factors for OS. The predictive power of TMEM79 for overall survival was evaluated using ROC curves that varied over time, and the statistical software packages "survival", "survivor", and "time ROC" were utilized for this analysis.

Univariate and multivariate analyses of TMEM79 and SMG5 are using R software. To establish a prognostic model for predicting OS in HCC, column line plots based on TMEM79 expression level, age, T stage, M stage, N stage, and clinical stage were constructed. Then, calibration curves for 1-year, 3-year, and 5-year survival rates were plotted to verify the consistency of OS data. Furthermore, through both univariate and multivariate analyses, it was confirmed that TMEM79 serves as an autonomous clinical characteristic for predicting OS. The "survival", "regplot" and "rms" packages of R software were used in this procedure.

### Functional phenotype analysis

Using R4.2.1 software, prognostic molecules associated with TMEM79 were studied. Circos plots were used to demonstrate the strong associations between TMEM79 and certain genetic markers. In addition, differential gene expression analysis was performed for the high and low expression groups to identify DEGs, with thresholds determined as |log FC|> 1 and FDR < 0.05. The DEGs were analyzed using KEGG enrichment analysis to uncover the molecular pathways and cellular processes associated with their enrichment. The R packages “limma”, “ggplot2”, “ggpubr”, “ggExtra”, “circlize”, “corrplot” and “pheatmap” were applied in this procedure.

### Immuno-infiltration analysis

Using "CIBERSORT", to assess the correlations between TMEM79 and SMG5 and immune cell infiltration in HCC, the expression profile of 22 immune cell subtypes was used to calculate the percentage of tumor-infiltrating immune cells. The differences in the levels of immune cell infiltration between high and low expression of TMEM79 and SMG5 were analyzed, and the results were demonstrated using box plots. Furthermore, scatter plots were utilized to illustrate the correlation between TMEM79 and SMG5 expression levels and immune cell infiltration, as determined by TIMER. Lollipop plots were also utilized to illustrate the association between immune cells and the expression levels of TMEM79 and SMG5. Analysis of the drug sensitivity of TMEM79 and SMG5 to CTLA4 and PD1. R software packages “reshape2”, “ggpubr”, “vioplot” and “ggExtra” were used in this procedure.

### Typing analysis of TMEM79-related molecules in HCC

Consensus Cluster Plus R program was used to classify individuals into discrete molecular clusters based on TMEM79 and their reciprocal gene expression. This has been accomplished using unsupervised clustering. In a Kaplan–Meier study, the clinical usefulness of the above gene in HCC was investigated using Kaplan–Meier method. We used survival and survivorship packages in R software to examine the survival curves and display the results. Later, principal component analysis was conducted using ggplot2 software. The biological processes of both subtypes were maintained using the gene set variation analysis tool. Malignant tumor tissue using expression and CIBERSORT were also used to represent the percentage of immune and stromal cells in patients with HCC. The extent to which each immune cell carried an enrichment score in each sample was also assessed by gene set enrichment analysis of individual samples.

### Immunohistochemistry

A tissue microarray (TMA) containing 282 cases was constructed using a 0.3 mm core. Each section was baked at 70 °C for approximately 1 h and dewaxed, followed by antigen repair with EDTA buffer (pH 9.0) for 20 min. After blocking endogenous peroxidase by adding 3% H_2_O_2_ for 15 min at room temperature, sections were incubated with TMEM79 rabbit polyclonal antibody (1:300 dilution; NOVUS; cat. no. NBP2-47601) at 4 °C overnight. After incubation with secondary antibody at room temperature for 30 min, the sections were stained with DAB assay. And then, after blocking endogenous peroxidase by adding 3% H_2_O_2_ for 15 min at room temperature, sections were incubated with SMG5 rabbit polyclonal antibody (1:150 dilution; ABCAM; cat. no. AB129107) at 4 °C overnight. Positive TMEM79 was brown and expressed in the membrane and cytoplasm of tumor cells, SMG5 was expressed in the nucleus. For TMEM79 and SMG5 staining, the intensity was scored as no staining (0), weak staining (1 +), moderate staining (2 +) or strong staining (3 +). The stained areas were classified into four categories based on the percentage (1) 0–25%, (2) 26–50%, (3) 51–75%, and (4) 76–100%. The staining intensity score and the percentage score were multiplied to obtain the final staining index. The analysis was performed independently by two professional pathologists, and high expression was considered when the combined score exceeded 6.

### Statistical analysis

The *t* test was employed to establish significant discrepancies in the expression of TMEM79 and SMG5 between HCC tissues and the adjacent normal liver tissues. The survival rates were determined by utilizing Kaplan–Meier survival curve analysis and a log-rank test. Correlation analysis was performed by Pearson's method, and Pearson’s correlation coefficient was performed by a two-tailed *t* test. A *p* value below 0.05 was considered statistically significant.

## Data Availability

The data sets used or analyzed during the current study are available from the corresponding author on reasonable request.
